# Indigenous *Pseudomonas* spp. Strains from the Olive (*Olea europaea* L.) Rhizosphere as Effective Biocontrol Agents against *Verticillium dahliae*: From the Host Roots to the Bacterial Genomes

**DOI:** 10.3389/fmicb.2018.00277

**Published:** 2018-02-23

**Authors:** Carmen Gómez-Lama Cabanás, Garikoitz Legarda, David Ruano-Rosa, Paloma Pizarro-Tobías, Antonio Valverde-Corredor, José L. Niqui, Juan C. Triviño, Amalia Roca, Jesús Mercado-Blanco

**Affiliations:** ^1^Department of Crop Protection, Institute for Sustainable Agriculture (CSIC), Córdoba, Spain; ^2^Bioinformatics Department, Sistemas Genómicos S.L., Valencia, Spain; ^3^Bio-Ilíberis Research and Development SL, Polígono Industrial Juncaril, Granada, Spain

**Keywords:** *Olea europaea*, verticillium wilt, biocontrol, *Pseudomonas*, rhizobacteria, *Pseudomonas indica*

## Abstract

The use of biological control agents (BCA), alone or in combination with other management measures, has gained attention over the past decades, driven by the need to seek for sustainable and eco-friendly alternatives to confront plant pathogens. The rhizosphere of olive (*Olea europaea* L.) plants is a source of bacteria with potential as biocontrol tools against Verticillium wilt of olive (VWO) caused by *Verticillium dahliae* Kleb. A collection of bacterial isolates from healthy nursery-produced olive (cultivar Picual, susceptible to VWO) plants was generated based on morphological, biochemical and metabolic characteristics, chemical sensitivities, and on their *in vitro* antagonistic activity against several olive pathogens. Three strains (PIC25, PIC105, and PICF141) showing high *in vitro* inhibition ability of pathogens' growth, particularly against *V. dahliae*, were eventually selected. Their effectiveness against VWO caused by the defoliating pathotype of *V. dahliae* was also demonstrated, strain PICF141 being the rhizobacteria showing the best performance as BCA. Genotypic and phenotypic traits traditionally associated with plant growth promotion and/or biocontrol abilities were evaluated as well (e.g., phytase, xylanase, catalase, cellulase, chitinase, glucanase activities, and siderophore and HCN production). Multi-locus sequence analyses of conserved genes enabled the identification of these strains as *Pseudomonas* spp. Strain PICF141 was affiliated to the “*Pseudomonas mandelii* subgroup,” within the “*Pseudomonas fluorescens* group,” *Pseudomonas lini* being the closest species. Strains PIC25 and PIC105 were affiliated to the “*Pseudomonas aeruginosa* group,” *Pseudomonas indica* being the closest relative. Moreover, we identified *P. indica* (PIC105) for the first time as a BCA. Genome sequencing and *in silico* analyses allowed the identification of traits commonly associated with plant-bacteria interactions. Finally, the root colonization ability of these olive rhizobacteria was assessed, providing valuable information for the future development of formulations based on these strains. A set of actions, from rhizosphere isolation to genome analysis, is proposed and discussed for selecting indigenous rhizobacteria as effective BCAs.

## Introduction

Cultivated olive (*Olea europaea* L. subsp. *europaea* var. *europaea*) is one of the most important oil crops in the world. It constitutes an agro-ecosystem of major relevance for the Mediterranean Basin since 90% of the global olive oil and table olive production is concentrated in this area (FAOSTAT, 2016) ^1^. Severe losses, and even tree death, are caused by a range of olive pathogens. Among them, the soilborne fungus *Verticillium dahliae* Kleb., causing Verticillium wilt of olive (VWO), represents a major threat in many regions where this tree is cultivated. Currently, however, no individual measure has proven effective to control VWO, and an integrated disease management strategy is therefore highly recommended (López-Escudero and Mercado-Blanco, 2011). Within this holistic framework, the development and implementation of sustainable and eco-friendly disease control measures is essential.

Plants have co-evolved with specific communities of microorganisms (i.e., the plant microbiome) that play crucial roles for the host's development and health (Berg et al., 2017). Moreover, many components of the plant-associated microbiome, particularly at the rhizosphere level, may constitute the first line of defense against soilborne pathogens (Weller et al., 2007). Hence, the plant rhizosphere constitutes an important, yet insufficiently explored, reservoir of microorganisms with antagonist ability against pathogens. In this sense, the isolation, identification, and characterization of microorganisms with biocontrol potential and able of colonize, endure, and be adapted to a complex niche such as the rhizosphere constitute an interesting disease management strategy. The utilization of biological control agents (BCAs) to suppress pathogens has been studied in several pathosystems involving woody plants (e.g., Pliego and Cazorla, 2012; Kalai-Grami et al., 2014), although it has been implemented to a lesser extent compared to herbaceous species, annual crops, and seedlings (Cazorla and Mercado-Blanco, 2016). Moreover, available information on the diversity and structure of microbial communities associated with woody plants is scant (e.g., Aranda et al., 2011; Zarraonaindia et al., 2015), particularly at the nursery propagation stage (Sun et al., 2014). Likewise, our understanding on woody plant-BCA-pathogen interactions are still limited, i.e., mechanisms underlying biological control, influence of environmental factors, effectiveness of BCAs, interaction between a BCA and the plant microbiome once the former is released, BCA colonization ability, or plant responses to the BCA are issues that still need to be studied in more detail (Cazorla and Mercado-Blanco, 2016). In this sense, omics technologies are contributing to enhance our understanding of these tripartite interactions (Massart et al., 2015). However, their implementation in woody plants is considerably lower than in herbaceous species (e.g., Mgbeahuruike et al., 2013; Gómez-Lama Cabanás et al., 2014; Martínez-García et al., 2015b).

Several studies have shown the potential, or even the true efficacy, of diverse beneficial microorganisms to suppress Verticillium wilts in different hosts (revised by Deketelaere et al., 2017), including olive (e.g., Aranda et al., 2011; Carrero-Carrón et al., 2016; Markakis et al., 2016). Among them, some *Pseudomonas* spp. strains are highly competent in colonizing the rhizosphere (Lugtenberg et al., 2001) and able to suppress the deleterious effects caused by different pathogens (Haas and Défago, 2005; Mercado-Blanco, 2015). Some strains of *P. fluorescens* and *P. putida* have thus been demonstrated as effective BCA against VWO (Mercado-Blanco et al., 2004; Prieto et al., 2009; Maldonado-González et al., 2015b) under different experimental conditions. Nevertheless, most of the studies related with biological control of Verticillium wilts have been conducted under controlled or gnotobiotic conditions. Indeed, BCA performance under field conditions is a scenario not frequently explored in biocontrol research, particularly with woody plants (Markakis et al., 2016). Related to this, it is crucial to understand the complex trophic interactions taking place between a newly-introduced BCA and the indigenous microbial community present in a target site, as well as the influence that diverse biotic and abiotic factors can exert to them, thereby conditioning the performance and effectiveness of the BCA. It seems therefore reasonably to isolate, identify, and characterize beneficial microorganisms from the niche where they will be eventually deployed, since they are theoretically adapted to the environmental conditions they will confront upon release.

Performing *in planta* assays under non-gnotobiotic conditions is therefore crucial to assess the effectiveness of a potential BCA since parameters such as competition for nutrients and space, colonization ability, time of inoculation, mode and site of application, etc., can be evaluated in a near-natural scenario. This is particularly relevant in plants with large root systems as olive. For instance, *P. fluorescens* PICF7 (Martínez-García et al., 2015b), a natural root endophyte of olive roots (Prieto and Mercado-Blanco, 2008), has been demonstrated to be an efficient BCA against *V. dahliae* (Maldonado-González et al., 2015b). However, strain PICF7 seems to require a direct contact with the pathogen to display effective biocontrol, as it was recently demonstrated using a split-root study system (Gómez-Lama Cabanás et al., 2017).

Selection of novel effective BCAs is mostly dependent on the pathosystem under study. The combination of diverse screening methods and the inclusion of the host plant into the screening assay is essential to select BCAs acting through diverse modes of action that are not mutually exclusive (i.e., induced resistance, antibiosis, competition, mycoparasitism, etcetera). In addition, it is necessary to have a comprehensive knowledge of potential traits involved in colonization efficiency of the target site, biocontrol performance, and plant growth promotion efficacy. This key information is currently aided by implementing genomics approaches which, in addition, also assist to discard the presence of potential undesirable traits (i.e., pathogenicity and/or virulence factors) for plants, animals, and/or humans, phenotypes that must be ruled out when aiming to novel BCA-based formulations.

Considering this framework, the main objective of this study was to implement a strategy to isolate, identify, and in-depth characterize novel BCAs from the olive root/rhizosphere. This approach is based on the following major steps: (i) assessment of both *in vitro* and *in planta* effectiveness against diverse pathogens, (ii) genotypic and phenotypic characterization including features associated with biological control and plant growth promotion, (iii) metabolic profiling to obtain useful information related to microhabitat adaptation as well as to assist in the future development of bioformulations, (iv) genome sequencing and *in silico* analyses to allow taxonomic identification and to acquire essential knowledge on both the presence of beneficial traits and the absence of undesirable and/or potentially-harmful genotypes, and (v) evaluation of their root adhesion and colonization abilities. We test the hypothesis that the root/rhizosphere from young olive plants produced at nurseries is already an important source of beneficial bacteria which are also adapted to the target niche where they display biocontrol activity against VWO.

## Materials and methods

### Assessment of the *in vitro* antagonism ability of selected olive rhizobacteria against diverse olive pathogens

A collection of olive (cv. Picual susceptible to *V. dahliae*, 1-year-old) rhizobacteria originating from different commercial nurseries located in Córdoba province (southern Spain) was previously generated and preliminary characterized (Ruano-Rosa et al., 2017). Bacterial isolates were tested against several olive pathogens, namely *Rosellinia necatrix* Rn320 and Rn400 (López Herrera and Zea Bonilla, 2007), *Phytophthora cinnamomi* CH1100 (kindly provided by Dr. C. López-Herrera, Institute for Sustainable Agriculture, IAS)*, Pseudomonas savastanoi* pv. savastanoi (Psv) PSS-3 (IAS culture collection) and NCPPB 3335 (Pérez-Martínez et al., 2007), *Colletotrichum nymphaeae* Col.114 (Moral and Trapero, 2012), *C. godetiae* Col.516 (kindly provided by Dr. A. Trapero-Casas, University of Córdoba, UCO), and *V. dahliae* Lebrija 1 (defoliating [D] pathotype) (kindly provided by Dr. F.J. López-Escudero, UCO) and *V. dahliae* V-249I (non-defoliating pathotype) (Collado-Romero et al., 2006). To determine the *in vitro* antagonist activity of olive rhizobacteria, dual cultures were performed in potato dextrose agar (PDA) and nutrient agar (NA) media plates. For pathogenic fungi-olive rhizobacteria interactions, microorganisms (i.e., 10 μl drops of either fungal propagules or bacterial cell suspensions) were inoculated simultaneously on agar plates at a distance of 2.8 cm one from each other, and then incubated at 25°C in the dark until the pathogen covered the distance between both microorganisms in the control plates (i.e., only inoculated with the pathogen). For Psv-olive rhizobacteria interactions, experiments were carried out by inoculating the potential antagonists over Psv lawns (OD_600_ = 0.1) and incubated at 28°C in the dark for 48 h. In the case of *in vitro* antagonism against *V. dahliae* isolates, olive rhizobacteria yielding the best results against this pathogen were further reassessed against the same D isolate. Individual drops of *V. dahliae* biomass (i.e., mycelium plus conidia) were plated in the middle of PDA plates, and four equidistant 10-μl drops of each tested bacteria were inoculated. One control plate with just a suspension of the pathogen biomass was included on each experiment. Antagonist activity (i.e., halos or inhibition zones) was then scored. The relative inhibition index was calculated according to the equation (Rc-Ra)/Rc, where Rc is the average radius of *V. dahliae* colony in the absence of antagonist bacterium and Ra is the average radius of *V. dahliae* colony in the presence of antagonist bacterium (four equidistant points). These experiments were performed twice with three biological replicates per each interaction and used media. Analysis of variance (ANOVA) for this parameter was carried out. Mean values were compared by the Tukey HSD Test at *P* = 0.05 using Statistix program (Version 10.0 for Windows. Analytical software 1985-2013).

### Verticillium wilt of olive biocontrol assays

Two independent experiments (I and II) were carried out under non-gnotobiotic conditions to test the biocontrol performance of three (PIC25, PIC105, and PICF141) selected olive rhizobacteria showing the highest *in vitro* inhibitory ability against *V. dahliae. Pseudomonas fluorescens* PICF7 was included as a reference since this strain is a well-known BCA against *V. dahliae* (Prieto et al., 2009; Maldonado-González et al., 2015b; Martínez-García et al., 2015b). One hundred and fifty olive plants (cv. Picual, 5-month old) originating from a commercial nursery located in Córdoba province were grown in pots (11 × 11 × 12 cm, one plant per pot), each containing 300 g of the potting substrate used in the nursery. Pots were randomly distributed in three blocks (15 plants per treatment) in a greenhouse under natural lighting and day/night temperature of 27/21°C. Bacteria were grown on Luria-Bertani (LB) agar plates at 28°C during 24–48 h. Bacterial cells were then scraped off from plates with 5 ml of MgSO_4_·7H_2_O 10 mM. For each bacterial treatment, inoculation was performed by adding 150 ml of a suspension of bacterial cells (1·10^8^ cfu·ml^−1^ in sterile MgSO_4_·7H_2_O 10 mM) per pot. Non-bacterized plants (control) were just drenched with 150 ml of sterile MgSO_4_·7H_2_O 10 mM. One week after inoculation with the bacteria, plants were challenged with *V. dahliae* V937I, an isolate representative of the D pathotype (Collado-Romero et al., 2006), by adding 150 ml per pot of a conidia suspension (1·10^6^ conidial/ml) prepared as previously described (Gómez-Lama Cabanás et al., 2017). Non-inoculated plants (control) were watered just with 150 ml of water. Disease development was assessed by scoring the severity of symptoms on a 0–4 scale according to the percentage of affected leaves and twigs (0, no symptoms; 1, 1–33%; 2, 34–66%; 3, 67–100%; 4, dead plant) (Maldonado-González et al., 2015b) twice a week during 90 day after pathogen inoculation. Areas under the disease progress curve (AUDPC) were calculated and analysis of variance (ANOVA) for this parameter carried out. Mean values were compared by the Fisher's protected LSD at *P* = 0.05 using Statistix program (Version 10.0 for Windows. Analytical software 1985-2013). Other parameters as Severity (S), Disease incidence (DI), Mortality (M), and Disease intensity index (DII) were also calculated for each treatment.

### Phenotypic characterization of olive rhizobacteria

In order to identify phenotypes associated with biological control and/or plant growth promotion in the three olive rhizobacteria eventually selected, assays aimed to evaluate protease (Naik et al., 2008), catalase (Holt et al., 1994), phosphatase (Katznelson and Bose, 1959; Naik et al., 2008), chitinase (Murthy and Bleakley, 2012), phytase (Hosseinkhani et al., 2009), celulase, xylanase, β-glucosidase, glucanase (Gong et al., 2012), pectinase (McKay, 1988), and amylase (Tindall et al., 2007) activities, as well as HCN (Bano and Musarrat, 2003), indole acetic acid (IAA) (Naik et al., 2008), 2,3-butanediol (MRVP medium, instructions according to Micro Media, Nebotrade Ltd.; Budapest, Hungary), and siderophore (Alexander and Zuberer, 1991) production were carried out. Production of the major siderophore pyoverdine was also investigated in liquid succinate mimimal medium (SSM; Meyer and Abdallah, 1978). Production of the typical green-yellow fluorescence in culture supernatants, related to the production of the siderophore pyoverdine (Dimkpa, 2016), was examined at 24, 48, and 72 h under UV light at 360 nm and by measuring the absorbance at 360–480 nm using a spectrophotometer. Finally, nutritional requirements of the selected strains were assessed by determining their ability to metabolize 71 different carbon sources, as well as their sensitivity to 23 chemicals, using the GEN III MicroPlate™ (Biolog, Hayward, CA) system according to the manufacturer's instructions. All these tests were repeated at least once.

### Molecular identification of genes commonly associated with biological control activity

Presence of *Pseudomonas* spp. specific genes involved in the biosynthesis of antibiosis-related compounds or associated with biocontrol phenotypes was performed by PCR analyses. Thus, gene-specific primers for the production of 2,4-diacetylphloroglucinol [DAPG], pyrrolnitrin [PRN], pyoluteorin [PLT], hydrogen cyanide [*hcnBC*], rhizoxin [*rzxB*], pyocin, and the insecticidal toxin protein [*FitD*] were used. Details on primer pair sequences as well as on amplification conditions are summarized in Supplementary Table S1. Amplifications were performed in a total volume of 50 μl containing 5 μl of 10 × PCR buffer (50 μM KCl, 10 mM Tris-HCl pH 9 [25°C], 1% v/v Triton X- 100), 1.5 mM MgCl_2_, 50 pmol each primer, 200 μM each dNTP, 0.5 U of Taq DNA polymerase (Roche®, Mannheim, Germany), and 25 ng of bacterial DNA. Amplification was performed in a DNA thermal cycler (Bio-Rad, Hercules, CA). Amplification products were separated by electrophoresis on a 1% agarose gels using 1x TAE buffer (Sambrook et al., 1989). For each PCR reaction, the reference strain *P. protegens* Pf5 was used as positive control for amplification of selected genes (Supplementary Table S1) (Kraus and Loper, 1992; Nowak-Thompson et al., 1994; Parret et al., 2005; Paulsen et al., 2005; Loper et al., 2008, 2016).

### Sequencing and bioinformatics analyses of olive rhizobacteria genomes effective against VWO

Bacterial DNA was obtained by using “JETFLEX Genomic DNA Purification Kit” (Genomed, Löhne, Germany) according to the manufacturer's instructions. The genomes of strains PIC25, PIC105, and PICF141 were sequenced following a high-throughput sequencing strategy by using an Illumina MiSeq (2015 Illumina, Inc.) system, paired-end technology and *de-novo* sequencing protocol at Sistemas Genómicos S.L. (Paterna, Valencia, Spain). The read size was 150 bp (300 bp for the paired reads) and the total initial reads were from 6,291,558 (lowest) to 9,873,454 (highest), giving a fold coverage of 157.06 to 255.05. The quality of the raw data was checked using FASTQC tools (http://www.bioinformatics.babraham.ac.uk/projects/fastqc/). All the adaptors were removed with the Fastq mcf (v1.04.803) (Aronesty, 2011) tool, and then a quality filter was made with Cutadapt (v1.9.1) (Martin, 2011) using a quality window value of 30. After the reads cleaning process the paired-end reads were merged using Flash (v1.2.11) (Magoč and Salzberg, 2011). With a masking step for the low quality bases, reads were ready to be assembled. Different assemblers were used but the main were Megahit (v1.0.3-29-g707d683) (Li et al., 2016) and Velvet (v1.2.10) (Zerbino and Birney, 2008). A list of several k-mers was used, from 71 to 99. Once the best assemblies were selected using the best N50 criteria, the annotation process started using Glimmer3 (Delcher et al., 1999; Kurtz et al., 2004) for the ORF detection and Blast V.2.2.30+ (Altschul et al., 1997) with an E-value cutoff of 1e-^3^ against the latest version (UniProtKB/Swiss-Prot Release 2015_08) of the Uniprot Swissprot protein curated database for bacteria (http://www.uniprot.org/). All the small local alignments were removed applying a filter requiring an alignment size of at least half size of the smallest sequence. All the sequences without a hit after removing the small local alignments were annotated using BLAST V.2.2.30+ (Altschul et al., 1997) against the last version (January 12, 2015) of the NT database (non-redundant nucleotide sequences from all traditional divisions of GenBank, EMBL, and DDBJ excluding GSS, STS, PAT, EST, HTG, and WGS) from the NCBI. Again, all the small local alignments were removed. Identified genes were functionally annotated using functional annotation of Uniprot (The UniProt Consortium, 2008) database from the previous step according to three different functional categories (biological process, molecular function, and cellular component). To allow a better understanding of the obtained genomes and their arrangements, each sample was aligned to the closest relative species available at NCBI (https://www.ncbi.nlm.nih.gov/) using Projector2 (Van Hijum et al., 2005). The new obtained pseudo assembly was processed again using Glimmer3 (Delcher et al., 1999) and Blast V.2.2.30+ (Altschul et al., 1997) against Uniprot (http://www.uniprot.org/). Genome sequences were deposited at Genbank under the accession IDs SAMN06276402, SAMN06276401, and SAMN06276230 for PICF141, PIC105, and PIC25, respectively.

In order to localize bacterial secretion systems (TSSs) machineries (T3SS, T4SS, and T6SS), a BLASTp and HMMER search was performed by using T346Hunter web application (Martínez-García et al., 2015a). Moreover, bioinformatics identification of genetic factors (i.e., adhesion, antibiotics, biofilm, detoxification, synthesis, and secretion of exopolysaccharides [EPSs], microbe-associated molecular patterns [MAMPs], multidrug resistance [MDRs], bacterial lipopolysaccharides [LPSs], plant cell wall-degrading enzymes [PCWDEs], phytohormones, phytotoxins, pigmentation, proteases, siderophores, etc.) involved in plant-bacteria interaction was performed by implementing the PIFAR open-access, web-based tool (Martínez-García et al., 2016).

Since strains PIC25 and PIC105 showed as highly similar and closest to *P. indica* species (see below), the data set containing ortholog alignments obtained by Glimmer3 as described above was used to compare the genomes of strains PIC25 and PIC105 and two recently-released, unpublished genomes of *P. indica* strains (i.e., JCM21544; Varghese, 2016, and NBRC 103045; Hosoyama, 2017) available in the NCBI database. A genome level comparison was performed in order to obtain the putative pangenome and coregenome of *P. indica*, as well as to identify exclusive genes present in each analyzed *P. indica* strains. Cd-hit-est (Fu et al., 2012) tool was used over the ORFs obtained from each sample with a homology level of 90%. The new clusters were annotated against Uniprot. After the annotation, pangenome, coregenome, and exclusive genes were extracted for our strains, and associated KEGG Ontology pathways (Kanehisa et al., 2017). Additionally, gene Ontology database (Ashburner et al., 2000) and PFam (Finn et al., 2009) terms for PIC25 and PIC105 were obtained to count and sort them in order to create a functional view of the differences.

### Verification on the absence of undesirable traits in the olive rhizobacteria of the *P. aeruginosa* group

To exclude the presence of pathogenicity/virulence-related genes in the genomes of strains PIC25 and PIC105, which clustered within the *P. aeruginosa* group (see below) that includes a number of pathogenic representatives, a genome level comparison was performed in order to obtain the pangenome, coregenome, and specific genes of PIC25 and PIC105 and two *P. aeruginosa* strains, one pathogenic, LESB58 (Winstanley et al., 2009) and another non-pathogenic, M18 (Wu et al., 2011). Besides, several virulence-related genes against mammals were also searched in the genomes of the selected olive rhizobacteria. These genes were present in the pathogenic *P. aeruginosa* strain LESB58 and absent, truncated or sharing less than 70% identity in the non-pathogenic strain *P. aeruginosa* strain M18 (Wu et al., 2011). Finally, we assessed pyocyanin (the major phenazine secreted by *P. aeruginosa*) production at 30/37°C after 30 days in liquid SSM, using *P. aeruginosa* BIRD-69 (Bio-Ilíberis collection) as positive control. Moreover, presence/absence of genes related to the synthesis of pyocyanin was *in silico* checked.

### Phylogenetic analysis of selected olive rhizobacteria

A multi-locus sequence analysis (MLSA) (7610 nt positions) was carried out using partial sequences of the following housekeeping genes: *16S rDNA* (1405 nt), *gyrB* (1566 nt), *atpA* (1496 nt), *nusA* (1349 nt), *recA* (995 nt), and *dnaJ* (799 nt) in order to assess the taxonomic position of *Pseudomonas* spp. strains PIC25, PIC105, and PICF141. Gene sequences were obtained from the genomes of the three olive rhizobacteria here sequenced, and compared with the corresponding sequences of 18 selected *Pseudomonas* spp. type strains (retrieved from different public databases, i.e., NCBI, EMBL, KEGG, etc.,) and phylogenetically related to the three strains under studied. A dendrogram was generated with TREECON for Windows software (Van de Peer and De Wachter, 1994), using the Neighbor-Joining algorithm. *P. entomophila* L48 was used as out-group species.

A second analysis was performed (1096 nt positions) using the partial sequences of the *gyrB* (496 nt) and *rpoD* (600 nt) genes, in order to identify the three newly-identified strains at the species level. To achieve this goal, sequences were compared with those of 33 *Pseudomonas* spp. type strains belonging to the closest *Pseudomonas* groups, and according to the results obtained from the above-mentioned analysis. For both trees, bootstrap analyses (1,000 replications) were performed. Distance matrixes were generated using the Maximum Composite Likelihood model (Tamura et al., 2004) with Molecular Evolutionary Genetics Analysis version 5 (Mega5) (Tamura et al., 2011) software, and percentages identity of the concatenated sequences were inferred with the Clustal 2.1 version.

### Root colonization assay

In order to evaluate the root colonization ability of the newly-selected olive rhizobacteria, bioassays were carried out using maize (*Zea mays* L.) seedlings as model plant and an experimental set-up previously described (Roca et al., 2013) with minor modifications. Bacterial strains were cultured overnight at 30°C in LB broth and culture turbidity was adjusted to OD_660_ = 1 in a final volume of 1 ml of M9 minimal medium (Sambrook et al., 1989). Maize seeds were surface sterilized by washing them twice with 70% (vol:vol) ethanol for 10 min, rinsed and washed again twice with 1% (vol:vol) bleach for 15 min and, finally, thoroughly rinsed with sterile deionized water. Seeds were then germinated on water-agar plates at 30°C for 2 days and overnight cultures that had been previously grown in LB broth were diluted in M9 to OD_660_ = 1. Next, germinated seeds were added to 2 ml of bacterial suspensions (5μl/ml of OD_660_ = 1 culture) of each strain tested. Seeds were then introduced in 50 ml skirted-base sterile tubes containing 35 ml of sterile sand, watered with 5ml of sterile deionized water and kept at room temperature for 2 weeks. To recover bacteria from the rhizosphere, stems were cut off, and roots were weighed and placed in sterile 50 ml screw-cap conical tubes containing 20 ml of M9 salts and 10–20 glass beads (2-mm diameter). The tubes were mixed by vortex for 2 min and the number of CFU per gram of root was determined for each plant by plating serial dilutions on LB medium. Bacterial colonization of maize roots over time was assessed by sampling roots at 3, 7, and 15 days. This assay was performed twice with three replicates per treatment and time-point.

Descriptive statistical analysis was performed. Mean and absolute errors of the data were calculated. Also inferential statistical analysis was done, specifically, analysis of variance (ANOVA), assuming a normal distribution of the data and homocedastacity. For *post-hoc* analysis, the Tukey test (*P* < 0.05) was used to determine differences in the ability of colonizing seeds. Statistical analysis was performed in R language for statistical computing (R Development Core Team, 2014).

## Results and discussion

### Assessment of olive rhizosphere bacterial strains as effective *in vitro* antagonists of relevant olive pathogens

In a preliminary study, a collection of bacterial isolates originating from the rhizosphere of nursery-produced olive plants showed *in vitro* antagonism against several relevant olive pathogens, including *V. dahliae* (Ruano-Rosa et al., 2017). Based on these results three of the most promising strains (namely PIC25, PIC105, and PICF141) were selected, and reproducibility and consistency of their antagonist behavior were corroborated (Table 1). Results showed that two strains (PIC25 and PIC105) antagonized the six pathogens tested (i.e., *V. dahliae, R. necatrix, P. cinnamomi, C. nymphaeae, C. godetiae*, and Psv). The only exception was *P. savastanoi* pv. savastanoi strain NCPPB 3335, that showed no growth inhibition regardless the tested medium or the olive rhizobacteria. In contrast, strain PICF141 (and the reference BCA *P. fluorescens* PICF7) only showed growth inhibition of three out of the six pathogens tested (*V. dahliae, P. cinnamomi*, and *C. godetiae*; Table 1). Differences were found depending on the culture medium used (PDA or NA), particularly for strain PIC105 for which differences were detected in five out of the nine assays performed (Table 1). Inhibition of pathogens' growth was more frequent in PDA for strains PIC25, PIC105, and PICF7, whereas strain PICF141 displayed higher antagonist activity in NA. Influence of culture media on results from *in vitro* antagonism tests has been previously reported by Trivedi et al. (2008), who showed that biomass reduction of pathogenic fungi *Alternaria alternata* (Fr.) Keissl. and *Fusarium oxysporum* Schltdl. by *Pseudomonas corrugata* Roberts and Scarlett. was dependent on the culture medium used. Thus, variable responses in reduction of fungal biomass were a consequence of the effect of specific nutritional factors on the antagonisms exerted by *P. corrugata*.

**Table 1:** *In vitro* antagonism assays against *Verticillium dahliae* and other olive pathogens.

**Pathogens**	**Vd Lebrija 1**		**Vd V-249I**		**Rn320**		**Rn400**		**Pc CH1100[A5]**		**Psv PSS3**		**Psv NCPPB 3335**		**Col.114**		**Col.516**	
**Media**	**PDA**	**NA**	**PDA**	**NA**	**PDA**	**NA**	**PDA**	**NA**	**PDA**	**NA**	**PDA**	**NA**	**PDA**	**NA**	**PDA**	**NA**	**PDA**	**NA**
***PSEUDOMONAS*** **STRAIN**																		
PIC25	+	+	+	+	+	+	+	+	+	−	+	−	−	−	+	+	−	+
PIC105	+	−	+	+	+	+	+	+	+	−	+	−	−	−	−	+	−	+
PICF141	+	+	+	+	−	−	−	−	−	+	−	−	−	−	−	−	−	+
PICF7	+	+	+	+	−	−	−	−	−	−	+	−	+	−	+	−	−	−

*Vd, Verticillium dahliae; Rn, Rosellinia necatrix; Pc, Phytophthora cinnamomi; Psv, Pseudomonas savastanoi pv. savastanoi; Col.114, Colletotrichum nymphaeae; Col.516, Colletotrichum godetiae. +, positive antagonism against the pathogen; – no antagonism. At least two biological replicates for each antagonism assay and culture medium were performed. PDA, Potato Dextrose Agar; NA, Nutrient Agar*.

The three olive rhizobacteria selected were able to significantly (*P* < 0.05) inhibit the growth of *V. dahliae* D pathotype, strain PIC105 showing the highest antagonistic capacity (relative inhibition index 0.428), followed by strains PIC25 (0.320) and PICF141 (0.174). These values were also significantly different among them. According to these results, the most promising strain to be used as BCA against VWO would be PIC105. Nevertheless, this potential benefit must be confirmed by conducting appropriate *in planta* experiments, since a range of biotic and abiotic factors present in the ecological niche where a BCA is deployed may greatly condition biocontrol effectiveness (Mercado-Blanco and Bakker, 2007). For instance, *P. fluorescens* PICF7 is an effective BCA against *V. dahliae* under different experimental conditions (Prieto et al., 2009; Maldonado-González et al., 2015a,b), although its *in vitro* antagonism ability was lower compared to *P. putida* strains also originating from the olive rhizosphere which, in contrast, did not show optimal VWO biocontrol performance (Mercado-Blanco et al., 2004). Also, Martín et al. (2015) reported that environmental conditions could affect the efficacy of preventive treatments with indigenous xylem endophytes in elm trees under field conditions, despite the fact these endophytes showed strong *in vitro* antagonism against the vascular pathogen *Ophiostoma novo-ulmi* Brasier. Moreover, biocontrol effectiveness can be critically influenced by the resident microbial communities (Errakhi et al., 2007; Goudjal et al., 2014). The *in vitro* tests performed only show the interaction between two microorganisms under very specific and artificial conditions. Since results could lead to erroneous assumptions concerning its true potential as BCA, effectiveness of a given BCA candidate must be demonstrated *in planta* and using experimental conditions resembling a natural scenario as much as possible. Thus, further VWO biocontrol experiments with selected olive rhizosphere strains were conducted under non-gnotobiotic conditions, avoiding major disturbance of the host rhizosphere.

### Strains PIC25, PIC105, and PICF141 are effective biocontrol agents of verticillium wilt of olive

Non-inoculated plants (control treatment) and plants inoculated only with bacterial strains showed normal development and growth in the two independent experiments performed (I and II). Neither abnormal appearance nor unexpected symptoms were observed during the bioassays. Five (experiment I) and three (experiment II) weeks after inoculation, first characteristic disease symptoms (including defoliation of green leaves) were observed in plants inoculated only with *V. dahliae* V937-I (disease control treatment). A more severe VWO syndrome was scored in experiment II than in bioassay I (Table 2). Approximately 1 week after the onset of the first symptoms in the disease control treatment, BCA/*V. dahliae* (Vd) treatments began to show the first VWO symptoms. Overall, pretreatment with the selected olive rhizobacteria reduced disease onset and development, either by reducing final DII or AUDPC. At the end of the experiment I all BCA/*Vd* treatments but PIC105/*Vd* showed a significantly (*P* < 0.05) lower AUDPC compared with the disease control treatment, though to a lesser extent than the disease suppressive effect exerted by strains PICF7 and PICF141. Compared with disease control plants, treatments with selected strains reduced all VWO parameters evaluated (final DI and DII, mortality and severity; Table 2). Regarding to experiment II, pretreatment with each of the strains significantly (*P* < 0.05) controlled VWO, even though disease pressure was much higher than in experiment I. Besides a significant reduction of the AUDPC, parameters such as final DII, mortality, and severity were also reduced in all BCA treatments compared to those observed in control treatment. The only exception was strain PIC105 that showed the same final DI than the disease control treatment (Table 2). Variability for some disease parameters observed between experiments highlights the need to conduct several independent assays to claim sound conclusions about the consistency of the suppressive effect of any BCA candidate. This is particularly relevant for tripartite interactions taking place in a complex niche as the rhizosphere. Moreover, in our opinion, results from each experiment should be shown separately (Mercado-Blanco et al., 2004; Maldonado-González et al., 2015a,b).

**Table 2:** Assessment of biocontrol activity of *Pseudomonas* spp. strains against Verticillium wilt of olive (defoliating pathotype).

**Treatments**	**Experiment I**					**Experiment II**				
	**Disease parameters^a^**					**Disease parameters^a^**				
	**AUDPC**	**Final DI (%)**	**Final DII**	**M (%)**	**S**	**AUDPC**	**Final DI (%)**	**Final DII**	**M (%)**	**S**
*V. dahliae*/PIC25	44.4**bc**	40	0.36	30	1.45	134.4**b**	100	0.89	80	3.58
*V.dahliae* /PIC105	53.8**abc**	50	0.49	40	1.95	97.1**bcd**	93	0.83	73.3	3.3
*V. dahliae* /PICF141	24.4**c**	26.6	0.24	13.3	0.96	65.1**cd**	73.3	0.59	40	2.36
*V. dahliae* /PICF7	19.9**c**	40	0.23	40	0.92	64.3**d**	66.6	0.56	40	2.25
*V. dahliae*	112.3**a**	70	0.61	60	2.42	174**a**	100	0.99	93.3	3.98

a *AUDPC, area under the disease progress curve over time. Final DI, final disease incidence (%). Final DII, disease intensity index ranging 0–1 was calculated with data on incidence and severity of symptoms recorded at 90 days. M, dead plants at the end of the experiment (%) (90 days). S, mean of disease severity symptoms at the end of the experiment (from 0 to 4). Data are the average of three randomly-distributed blocks each with five pots per treatment. Control (non-inoculated) plants did not show any disease symptoms and were not included in the statistical analysis. Means in a column followed by different letters are significantly different according to Fisher's protected LSD test (P = 0.05)*.

The three newly-identified indigenous olive rhizobacteria were thus able to reduce significantly disease severity, highlighting strain PICF141 with a comparable biocontrol performance to that previously demonstrated for the reference strain *P. fluorescens* PICF7 (Prieto et al., 2009; Maldonado-González et al., 2015b). Since these results do not correlate with *in vitro* antagonist assays, *in planta* experiments must be always performed to unequivocally demonstrate biocontrol activity. Indeed, while these tests provide useful information related to antibiosis and/or competition for nutrients as mode of actions of a given BCA, they clearly overlook other mechanisms only operating in the target niches, such as induction of resistance and/or competition for space. Considering this outcome these newly-isolated olive rhizobacteria can be postulated as effective BCA against VWO and be further studied at phenotypic and genomic levels. Basic knowledge from these analyses will be instrumental for the future development of single- or consortia-based bioformulations.

### Identification of plant growth promotion and biocontrol activities

All strains were positive for phytase and catalase activities, as well as for siderophore(s) production although only one of the three strains tested (strain PICF141) was positive for the production of the siderophore pyoverdine, in contrast to data from *in silico* analysis (see below). Phytases are phosphatases catalyzing the hydrolysis of phytic acid, thereby releasing a usable form of inorganic P for the plants. Bacteria with phytase activity have been isolated from the rhizosphere and proposed to promote plant growth in soils with high content of organic P (Singh et al., 2014). Studies have revealed that phytase-producing rhizobacteria not only harbor the ability to mineralize phytate but also harbor other PGPR activities, such as the production of indole acetic acid, siderophore, volatiles, and ammonia (Saharan and Nehra, 2011). None of them were positive for production of the volatile 2,3-butanediol nor for pectinase and chitinase activities (Table 3). Very slight xylanase and glucanase activities were detected only for *P. fluorescens* PICF7. Strain PICF141 was the only strain showing β-glucosidase activity and HCN production (Table 3). The production and release of HCN by beneficial rhizobacteria has been studied as a biocontrol mechanism (i.e., antibiosis) displayed by a number of BCAs. Examples of the inhibitory effect of bacterially-produced HCN have been shown for fungi (Voisard et al., 1989), plants (Alström and Burns, 1989), nematodes (Gallagher and Manoil, 2001), insects (Devi et al., 2007), and other bacteria (Rudrappa et al., 2008). Strains of different *Pseudomonas* spp. are known to be cyanogenic, exerting toxic effects against various prokaryotes and eukaryotes even if they are not in close contact (Cernava et al., 2015). In addition, a role in increased phosphate availability for rhizobacteria and plant hosts has also been proposed for HCN (Rijavec and Lapanje, 2016). Production of HCN by PICF141 further supports its use as a broad-range BCA, antibiosis being one of the predicted modes of action for this rhizobacteria. While this fact correlated with the performance of strain PICF141 as BCA of VWO, PICF141 did not show broad *in vitro* antagonism against olive pathogens, in contrast with strains PIC25 and PIC105 which inhibited most of the pathogens analyzed (Table 1).

**Table 3:** Phenotypic characterization of *Pseudomonas* spp. strains of properties associated with plant growth promotion and/or biocontrol.

**Isolates**	**Activities**														
	**Siderophores**	**Fosfatase**	**Phytase**	**AIA**	**Protease**	**Cellulase**	**Pectinase**	**Chitinase**	**B-Glucosidase**	**Amilase**	**Xilanase**	**Glucanase**	**HCN**	**Catalase**	**2,3-Butanediol**
PIC25	+	−	+	+	−	+	−	−	−	+	−	−	−▪	+	−
PIC105	+	±	+	+	−	+	−	−	−	+	−	−	−▪	+	−
PICF141	+	±	+	+	+	−	−	−	+	−	−	−	+	+	−
PICF7	+	+	+	−	+	+	−	−	−	−	±	±	−	+	−

*+, presence of activity; −, absence of activity; ±, ambiguous result; ▪, isolate did not growth in King's B medium. Results shown were obtained from LB cultures amended with L-glycine. At least two biological replicates for each activity/strain were performed*.

Results from the Biolog GENE III microplates showed that strains PIC25 and PIC105 shared most of the properties tested (89/95) (i.e., biochemical properties, utilization of carbon sources, chemical sensibilities) differing only in a few metabolic characteristics (Supplementary Table S2). For instance, strain PIC25 was able to utilize sodium butyrate, α-keto-butyric acid, D-frutose-6-PO4, D-serine, whereas strain PIC105 was unable to utilize any of these substrates but could use quinic acid. PIC25 and PIC105 were able to grow in the presence of 2.5% NaCl, D-mannitol, L-arginine, L-histidine, L-malic acid, butyric acid, sucrose, D-fructose, and glycerol. Compared to the other strains, strain PICF141 clearly differed in its ability to use carbon sources as well as in its sensitivity to chemicals. In fact, PICF141 shared less than 70% of properties tested with all strains studied (Supplementary Table S2). Moreover, PICF141 was the only strain able to grow in D-maltosa and D-turanose. Finally, strain PICF141 was able to assimilate D-trealose, sucrose, α-D-glucose, D-fructose, D-galactose, myo inositol, glycerol, and L-serine.

An in-depth knowledge on the ability to use different carbon sources and chemical sensitivities, and optimum culturing medium among others, provides relevant information for the future formulation, production, storage, application, and commercialization of BCAs. The carbon substrate has a dual role in biosynthesis and energy generation, carbohydrates being the usual carbon source for microbial fermentation processes (Costa et al., 2002). Competition for carbon sources can be one of the main factors determining biocontrol efficacy when bioformulations are based on consortia of bacteria. This fact must therefore be thoroughly considered when developing multi-strain combinations (Sun et al., 2015).

### Phylogenetic analyses of newly-isolated olive rhizobacteria

Preliminary molecular identification (partial sequencing and comparison of *16s rRNA* and *gyrB* genes) (Ruano-Rosa et al., 2017) of the three selected strains enabled their assignment to the highly-ubiquitous and metabolically-versatile *Pseudomonas* genus. This genus comprises 238 species and 18 subspecies according to the List of Prokaryotic Names (http://www.bacterio.net/pseudomonas.html) (June, 2017). The sequence of the *gyrB* gene suggested *P. indica* as the closest species to strains PIC25 and PIC105 (e-values 0.0, and 93 and 98% identity, respectively), while strain PICF141 showed as closely related to *P. fluorescens* (e-value 0.0, and 95% identity). *Pseudomonas indica* was proposed as novel species by Pandey et al. (2002). The first strain of this species was isolated from an oilfield in Gujarat (India).

To more accurately identify the three novel *Pseudomonas* spp. strains a MLSA (7610 nucleotide positions from partial sequences of the housekeeping genes *atpA, dnaJ, gyrB, nusA, recA*, and *16S rRNA*) was carried out. The sequences of these housekeeping genes were obtained from their genomes (see below). Phylogenetic trees were generated including 21 *Pseudomonas* spp. type strains belonging to 13 different species and strains PIC25, PIC105, and PICF141 strains (Supplementary Figure S1). *Pseudomonas* is a rather complex genus subjected to continuous taxonomic revisions (Gomila et al., 2015), its heterogeneity being significantly resolved by polyphasic taxonomic studies (Achouak et al., 2000), *16S rRNA* gene sequence identity (Anzai et al., 2000), DNA-DNA hybridization relatedness (Palleroni, 1984), and MLSA (Mulet et al., 2010). According to the latter study, the genus was divided into two main intrageneric groups (IG), namely IG *P. aeruginosa* and IG *P. fluorescens*. The first IG includes three main groups represented by the species *P. aeruginosa, P. stutzeri*, and *P. oleovorans*, while the other IG comprises six main groups represented by the species *P. fluorescens, P. syringae, P. lutea, P. putida P. anguilliseptica*, and *P. straminea*, amongst which the *P. fluorescens* group is the most complex (Mulet et al., 2010). According to this classification the novel olive rhizobacteria strains were assigned to the *P. mandelii* subgroup within the *P. fluorescens* group (strain PICF141) and to the *P. aeruginosa* group (strains PIC25 and PIC105, that showed 96.47% identity between them) (Supplementary Figure S1). These results were consistent with our preliminary molecular identification only based on the comparison of the *16S RNA* and *gyrase B* genes (Ruano-Rosa et al., 2017). The closest neighbor of strains PIC25 y PIC105 was *P. resinovorans* type strain (89 and 89.59% identity, respectively). Strain PICF141 clustered within the *P. fluorescens* group, *P. mandelii* JR-1 strain being the closest species (94.47% identity).

**Figure 1:**
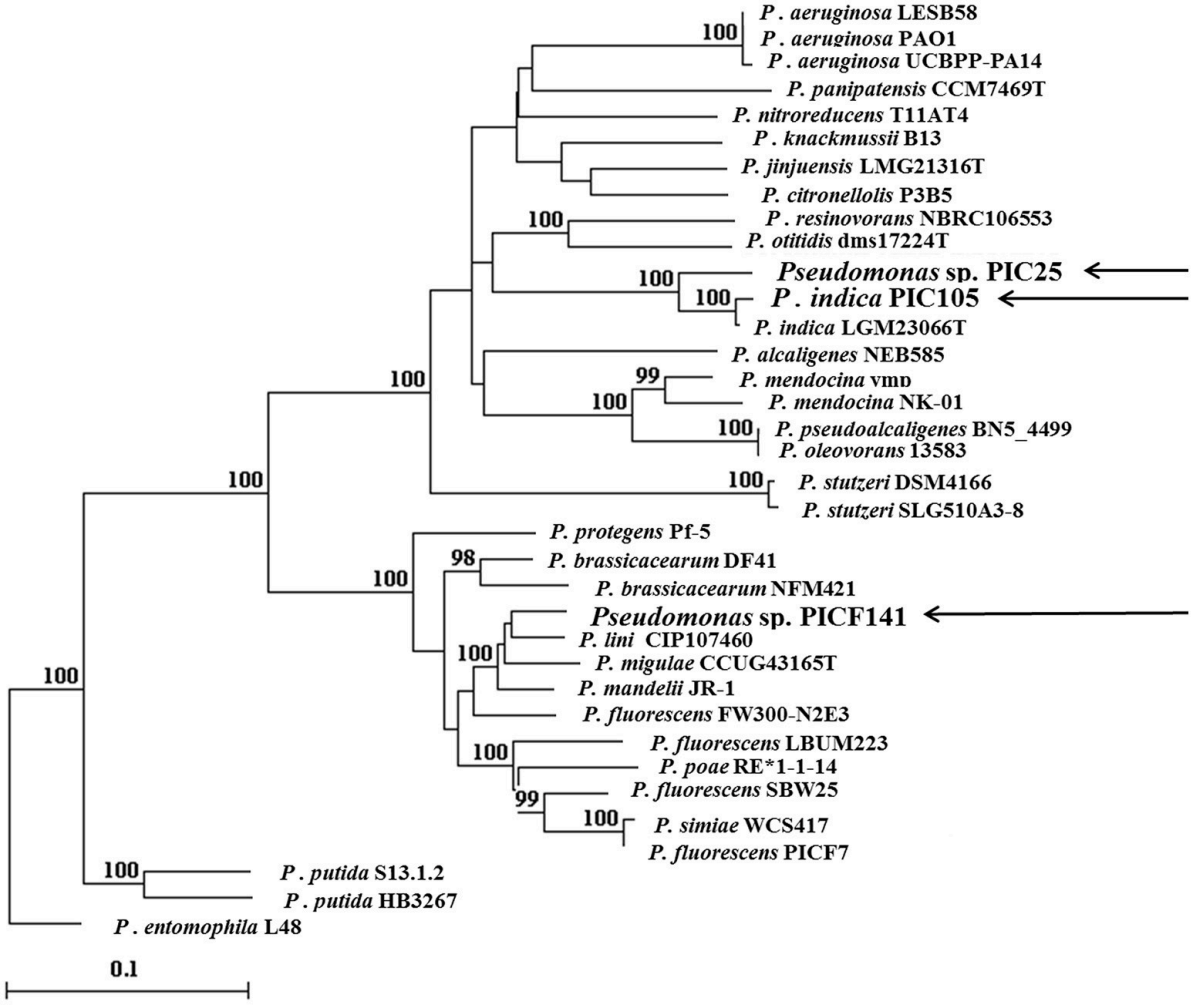
Phylogenetic tree showing the taxonomic position of the three new *Pseudomonas* spp. strains (arrowed) isolated from the olive rhizobacteria. The tree was inferred by the Neighbor-Joining method, based on the alignment of concatenated partial sequences of *gyrB* and *rpoD* genes (see text for details). Bar indicates sequence divergence. Bootstrap values (>95%) based on 1,000 re-sampled datasets are shown at branch nodes. *Pseudomonas entomophila* L48 was used as out-group.

For a better fine tuning taxonomic identification of the novel BCAs, additional MLSA (1096 positions of partial sequences of the housekeeping genes *gyrB* and *rpoD* gene) was conducted by comparing representative strains of the *P. fluorescens, P. mandelii, P. aeruginosa, P. oleovorans*, and *P. stutzeri* subgroups (33 *Pseudomonas* type strains belonging to 24 different species) (Figure 1). Results were congruent with the previous analysis, and strains PIC25 and PIC105 clustered within the *P. aeruginosa* group while strain PICF141 did so within the *P. mandelii* subgroup. This analysis indicated that the closest species to strains PIC25 and PIC105 was *P. indica* (Pandey et al., 2002), showing 94.24 and 98.63% identities, respectively. Moreover, the evolutionary distances separated *P. indica* type strain from either PIC25 (0.060) or PIC105 (0.014). Considering these results we assign strain PIC105 to the *P. indica* species. Despite the fact that PIC25 and PIC105 showed 96.47% identity and an evolutionary distance of 0.061, strain PIC25 was kept as *incertae sedis* within the *P. aeruginosa* group until further evidence, although showing close relatedness with *P. indica*. Indeed, PIC25 and PIC105 were able to grow in the presence of 2.5% NaCl, D-mannitol, L-arginine, L-histidine, L-malic acid, and butyric acid (Supplementary Table S2), as previously reported for *P. indica* IMT37 and IMT70 (Pandey et al., 2002). In addition, PIC25 and PIC105 strains shared with IMT40 strain the ability to grow in sucrose, D-fructose and glycerol.

Finally, strain PICF141 was also kept as *incertae sedis* within the *P. mandelii* subgroup, *P. lini* (Delorme et al., 2002) being the closest relative (95.49% identity and 0.041 evolutionary distance) (Figure 1). Interestingly, strain PICF141 and *P. lini* CFBP 5737T shared the ability to metabolize D-trealose, sucrose, α-D-glucose, D-fructose, D-galactose, myo inositol, glycerol, and L-serine (Supplementary Table S2; Delorme et al., 2002). In contrast, PICF141 was able to grow in D-maltosa and D-turanose (Supplementary Table S2) while its closest species was unable to use these two compounds as carbon source (Delorme et al., 2002).

### General characteristics of the PIC25, PIC105, and PICF141 genomes

A summary of the genome sequencing projects for strains PIC25, PIC105, and PICF141 is shown in Supplementary Table S3. The draft genomes of PIC25 (predicted size 6,053,123 bp; GC content 63.6%), PIC105 (5,806,705 bp; 64.2%), and PICF141 (6,008,661 bp; 58.8%) included 79, 69, and 57 large mapped contigs (largest contig sizes were 169,273, 252,661, and 300,602 nt, respectively). Non-mapped contigs were 36 (for PIC25), 33 (PIC105), and 14 (PICF141). Additional genome characteristics such as predicted number of coding sequences, number of rRNA operons, etc, are summarized in Table 4. The classification of coding DNA sequences into functional categories according to the COG (Clusters of Orthologous Groups) is shown in Supplementary Table S4.

**Table 4:** General parameters of the three olive rhizosphere *Pseudomonas* spp. genomes.

**Attribute**	**Genome (total)**					
	**PIC25**		**PIC105**		**PICF141**	
	**Value**	**% of total**	**Value**	**% of total**	**Value**	**% of total**
Genome size (bp)	6,053,123	100	5,806,705	100	6,008,661	100
DNA coding region (bp)	5,295,441	87.5	5,076,306	87.4	5,216,964	86.8
DNA G+C content (bp)	3,849,786	63.6	3,726,163	64.2	3,531,891	58.8
Total genes	5,779	100	5,326	100	5,462	100
Protein-coding genes	5,585	96.6	5,120	96.1	5,245	96.0
RNA genes	194	3.4	206	3.9	217	4.0
Genes in internal clusters	NA	–	NA	–	NA	–
Protein-coding genes with function prediction	3,369	58.3	3,572	67.1	3,872	70.9
Protein-coding genes assigned to COGs	2,737	47.4	2,744	51.5	2,907	53.2
Proteins with signal peptides	NA	–	NA	–	NA	–
Proteins with transmembrane helices	NA	–	NA	–	NA	–
CRISPR repeats	3	0.0018	5	0.0022	0	0.0000

### Comparative analysis of the PIC25 and PIC105 genomes with *P. indica* species

**Figure 2:**
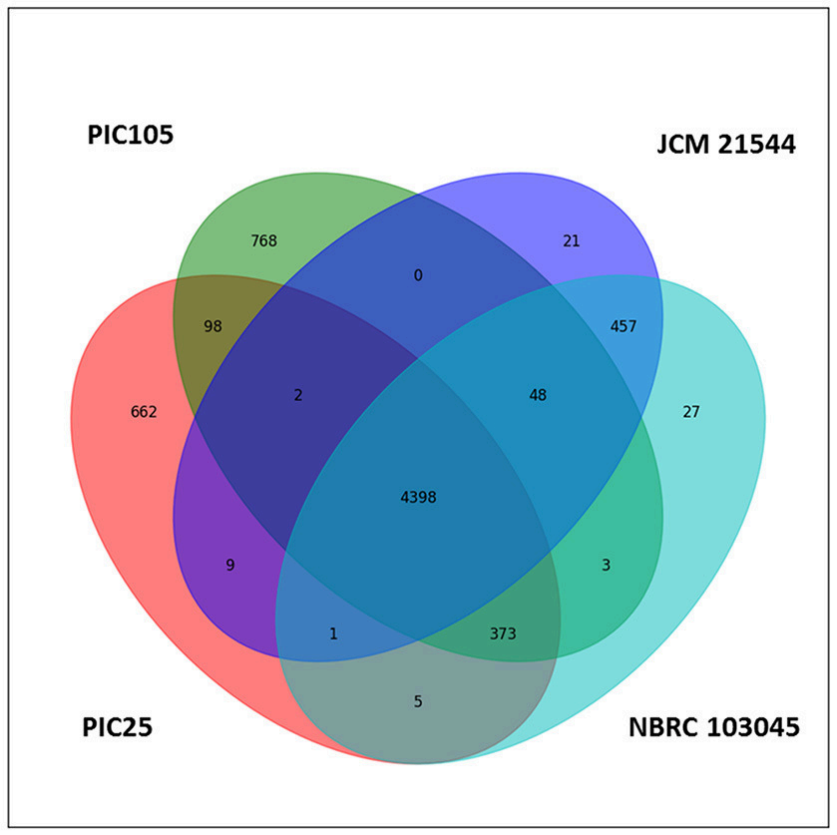
Comparison between the genomes of *Pseudomonas* sp. PIC25 and *Pseudomonas indica* PIC105, JCM21544, and NBRC 103045. The Venn diagram shows the number of orthologous coding sequences shared by the four strains (4398 genes; core genome) and those ones that are specific for PIC25 (662) and PIC105 (768).

Since strains PIC25 and PIC105 displayed considerable phylogenetic, phenotypic, and metabolic similarities between them, and that their closest species was *P. indica*, their genomes were compared with the draft genomes of two recently-sequenced *P. indica* strains, JCM21544 and NBRC 103045, available in the databases (https://www.ncbi.nlm.nih.gov/genome/?term=Pseudomonas+indica). The bioinformatics analysis enabled the identification of a putative *P. indica* core genome consisting of 4398 predicted protein coding genes (Figure 2). This core genome represented 79.3% (PIC25) and 77.3% (PIC105) of the predicted proteome of the olive rhizobacteria under study. A total of 662 genes were specific for PIC25 and 768 for PIC105. When analyzed PIC25 and PIC105 strains together, the core genome for the olive rhizobacteria consisted of 4871 putative protein-coding genes, while 677 and 819 predicted protein coding genes were unique for PIC25 and PIC105, respectively. Whilst both strains share a large number of genes with the two *P. indica* strains so far sequenced, the analysis carried out was not conclusive enough to claim that all strains can be accurately assigned to the same species; that is, to *P. indica*. Additionally, a functional comparison of PIC25 and PIC105 strains was made using the Gene Ontology terms associated to the strain-exclusive genes. No significant functional deviation was found. The unique GO term showing significant difference was the GO:0000746 P:conjugation (Biological Process category): 3.44% (PIC105) vs. 0% (PIC25). Another term (Biological Process category too) showing differences was the GO:0009405 P:pathogenesis, that was slightly more present in PIC25 (3.67 vs. 2.21%) (Supplementary Tables S5–S8).

### Ruling out the presence of potential pathogenicity/virulence factors in strains PIC25 and PIC105

Considering that strains PIC25 and PIC105 belong to the IG *P. aeruginosa*, and that some representatives of the *P. aeruginosa* species can behave as opportunistic pathogens in immuno-compromised patients (Stover et al., 2000), it is essential to discard that the new olive rhizobacteria harbor pathogenic traits and/or virulence factors. To this end, the genomes of strains PIC25 and PIC105 were compared with the genomes of two *P. aeruginosa* strains, one non-pathogenic (M18, Wu et al., 2011) and another pathogenic (LESB58, Winstanley et al., 2009). Even though strains PIC25 and PIC105 clustered within the *P. aeruginosa* group, the analysis revealed that they only shared 40 genes with strain LESB58 and 43 genes strain M18 (at >90% identity) (data not shown). Besides, none of the virulence-related genes (e.g., *flgL, fliC, flaG, fliD, fliS, fliT*, etc.,) against mammals present in LESB58 or M18 strains according to Wu et al. (2011) were found in the genomes of PIC25 and PIC105. Another characteristic feature of *P. aeruginosa* species is the production of the blue pigment pyocyanin (Wilson et al., 1988). Pyocianin is a phenazine exerting toxic effects on eukaryotic cells through reactive oxygen species (Mahajan-Miklos et al., 1999). The presence of pyocyanin biosynthesis genes was also checked in PIC25 and PIC105 genomes. Indeed, *PhzA1B1C1D1E1F1G1* and *phzA2B2C2D2E2F2G2* gene clusters, involved in the final steps of the pyocyanin biosynthetic cascade (Mavrodi et al., 2001), were not found in PIC25 and PIC105 genomes. The enzyme anthranilate synthase participates in the synthesis of pyocyanin. *Pseudomonas aeruginosa* possesses two functional anthranilate synthases, each comprised of large and small subunits encoded by the products of the *trpE* and *trpG* and *phnA* and *phnB* genes, respectively. These enzymes are not functionally redundant (Essar et al., 1990a,b). Only *TrpE* and *trpG* genes were identified in PIC25 and PIC105 genomes. Besides, pyocyanin production was tested at both 30 and 37°C. Results showed that after 30 days of culturing in liquid SSM, PIC25, and PC105 did not produce pyocyanin, in contrast to the reference strain *P. aeruginosa* BIRD-69 (data not shown). Altogether, these results indicate that strains PIC25 and PIC105 do not seem to carry undesirable traits present in some pathogenic representatives of the *P. aeruginosa* group, ruling out potential risks as for human toxicology concerns (Kamilova et al., 2015).

### *In silico* identification of secretion systems T3SS, T4SS, and T6SS

Bacterial secretion systems (TSSs) play relevant roles throughout the whole range of plant-bacteria interactions (i.e., pathogenic, endophytic, or mutualistic symbioses) (Costa et al., 2015; Green and Mecsas, 2016). The presence of TSSs in the genomes of strains PIC25, PIC105, and PICF141 was confirmed by using the “T346Hunter” web-based tool (Martínez-García et al., 2015a). “T346hunter” allowed the identification of flagellar and non-flagellar type III (T3SS), type IV (T4SS), and type VI (T6SS) secretion systems, although differences were found among the olive rhizobacteria under study. Moreover, presence of some of these TSSs in the reference strain *P. fluorescens* PICF7 was confirmed, corroborating our previous results for this BCA (Martínez-García et al., 2015b). The three TSSs were identified in the genomes of PIC25 and PIC105, while in PICF141 and PICF7 only T3SS and T6SS were detected. Finally, flagellar T3SS2 and 3, and T6SS1 and 2 were present in all strains (Supplementary Table S9). Overall, strains PICF7 and PICF141 showed a very similar TSSs profile, only differing in that PICF141 harbors flagellar T3SS1 while strain PICF7 has the non-flagellar T3SS1 (Supplementary Table S9).

Non-flagellar T3SS (NF-T3SS) and T6SS are complex molecular machineries that deliver effector proteins from bacterial cells into the environment or into other eukaryotic or prokaryotic cells, with significant implications for the strains encoding them. T3SSs have been detected in both beneficial and pathogenic bacteria, and have been related to plant root colonization, rhizosphere competence, environmental competition, defense against amoebas, or oomycete suppression (Rezzonico et al., 2005; Matz et al., 2008; Mavrodi et al., 2011). For instance, T3SSs have been identified in the PGPR *P. fluorescens* SBW25 (Preston et al., 2001) and BBc6R8 (Cusano et al., 2011), and in *P. brassicacearum* Q8r1-96 (Mavrodi et al., 2011). Strain Q8r1-96, as well as *P. fluorescens* A506, Q2-87, SS101, SBW25, and *Pseudomonas* sp. BG33R, harbor gene clusters encoding for secretion protein T3SS (*rsp/rsc*). In the pathogenic species complex *P. syringae*, T3SS is responsible of the transport of type III effector proteins (T3Es) into plant cells (Cornelis, 2010), involved in the suppression of plant defense responses and alteration of the eukaryotic cell physiology (Chang et al., 2004; Lindeberg et al., 2008). T6SSs, also present in the three *Pseudomonas* spp. here selected as BCAs, were originally thought to be exclusively implicated in the delivery of virulence effectors to eukaryotic hosts. However, other reports have indicated that this TSS play a key role in the interaction among bacteria, being well-distributed among environmental bacteria including plant-associated *Pseudomonas* spp. (Russell et al., 2011; Loper et al., 2012). Thus, one to three gene clusters coding for T6SS are present in the genomes of *P. fluorescens* group strains analyzed by Loper et al. (2012). Other beneficial pseudomonads like *P. putida* W619 also harbors, among other TSS, several T6SSs (Reinhold-Hurek and Hurek, 2011).

T4SS is a more functionally-diverse TSS system not only involved in effector translocation but also in conjugation and DNA uptake/release. There are three functional types of T4SSs (Alvarez-Martinez and Christie, 2009; Wallden et al., 2010). One of them is used for the conjugation process, which is the major mechanism to spread antibiotic resistance genes among pathogenic bacteria. Others T4SSs are involved in DNA uptake (transformation) and release from the extracellular milieu. Finally, there are T4SSs that are used to transfer proteins. Most of the T4SSs in this category are found in pathogenic bacteria, playing important roles in virulence such as establishing pathogen-host interaction and/or transferring toxic effector proteins or protein complexes into the cytoplasm of the host cell. While in strain PIC105 only T4SS1 was found, the three T4SS types were detected in the PIC25 genome. Their functionality and roles remain to be elucidated.

### *In silico* detection of genetic traits involved in bacteria-plant interaction

The “PIFAR” open-access web-based tool (Martínez-García et al., 2016) allowed the identification of several genetic factors involved in plant-bacteria interactions. A summary of the genes identified in the three newly-identified strains, as well as in *P. fluorescens* PICF7, is provided in Supplementary Table S10. Specific information about genetic factors can be found in Table 5. The number of annotated genes involved in plant-bacteria interaction present in strains PIC25, PIC105, and PICF141 was very similar, ranging from 108 to 116 (Supplementary Table S10). However, this number was lower than that detected in *P. fluorescens* PICF7 (>150 genes). The same gene clusters were identified in the genomes of strains PIC25 and PIC105 for factors such as proteases, detoxification, EPSs, LPSs, MDRs, volatiles, and MAMPs, confirming the close relatedness of these two bacterial strains. As expected, the profile of genetic factors in strain PICF141 differed significantly from that in PIC25 and PIC105, showing more similarities with strain PICF7 and in agreement with their assignment to the *P. fluorescens* group (Table 5 and Supplementary Table S10). For instance, less adhesion and EPS factors were found in strains PIC25 and PIC105 compared to strains PICF7 and PICF141. In contrast, more PCWDE and LPS factors, and particularly T3Es, were identified in strains PIC25 and PIC105 (Table 5 and Supplementary Table S10). Production of 2,3-butanediol was not detected in any of the studied strains. Biosynthesis of 2,3-butanediol from pyruvate requires three key enzymes, α-acetolactate synthase (ALS, EC 4.1.3.18), α-acetolactate decarboxylase (ALDC, EC 4.1.1.5), and 2,3-butanediol dehydrogenase (BDH, EC 1.1.1.76; also called acetoin reductase, EC 1.1.1.4) (Ji et al., 2011). Only the gene putatively coding for α-acetolactate synthase was found, what could explain the absence of 2,3-butanediol production. The *in silico* analysis also confirmed that the genomes of the three newly-identified strains harbor the genes encoding for the biosynthesis of several antibiotics (e.g., fusaricidin and amphisin). In contrast, genes coding for the insecticidal fit toxin and for HCN production were only identified in strain PICF141. *In vivo* production of HCN by this strain was previously confirmed, as well as the presence of *hcnBC* genes by PCR analysis (see above).

**Table 5:** Genes identified in the genomes of *Pseudomonas* spp. strains PIC25, PIC105, PICF141, and PICF7 involved in plant-bacteria interaction according to the web-based tool PIFAR (Martínez-García et al., 2016).

**Genetic factors involved in plant-bacteria interaction**		***Pseudomonas*** **spp. strains**			
		**PIC25**	**PIC105**	**PICF141**	**PICF7**
Proteases	*HtrA*	+	+	+	+
	Serralysin protease C	+	+	+	+
PCWDEs	*LipA*	+	+	+	+
	Pec lyase C	+	+	−	−
	Pectate lyase	+	+	−	−
	Cellulase		+	+	+
Adhesion	*AttC*	+	+	+	+
	*AttG*	−	−	+	+
	*XadM*	−	−	+	+
	Haemagg act	+	+	+	+
	Usher	−	−	+	+
	Pilin	+	−	+	+
	Fimbrial	−	−	+	+
	Cellulose synthase	−	−	+	−
Deto+ification	*SapABCDF*	+	+	+	+
	*KatB*	+	+	+	+
	*KatE*	+	+	+	+
	*KatG*	−	−	+	−
	*Pip*	+	+	+	+
	*Dps*	+	+	−	+
	*Cbb*	+	+	+	+
	Copper resistance cueAR	+	+	+	+
	Copper resistance ABCDRS	−	−	+	+
	Polymixin resistance	−	−	−	+
EPSs	Alginate	+	+	+	+
	*GaIU*	+	+	+	+
	*GpsX*	−	−	+	+
Metabolism	*TrpGC*	+	+	+	+
	Glutamate synthase gltBD	+	+	+	+
	*AroC*	+	+	+	+
	*AroK*	+	−	+	+
	*AroQ*	+	+	+	+
	*AcnB*	+	+	+	+
	*AsnB*	+	+	+	+
	*Mqo*	+	+	+	+
	Purine biosynthesis *purD*	+	+	+	+
	Purine biosynthesis *purC*	+	+	−	−
	Citrate transporter	−	−	−	+
LPSs	Wzt	+	+	+	+
	Rfb303	+	+	+	+
	Glicosyl tranferase WxocB	+	+	−	−
MDRs	ACR tran	+	+	+	+
	Multi Drug Res	+	+	+	+
	*MatE*	+	+	+	+
	*OEP*	+	+	+	+
Volatiles	*BudB*	+	+	+	+
	2,3-butanediol	+	+	+	+
Antibiotics	Fusaricidin	+	+	+	+
	Amphisin	+	+	+	−
	Hydrogen cyanide	−	−	+	−
	Insecticidal fit toxin	−	−	+	−
	Viscosin	−	−	−	+
	Gluconic acid	−	−	−	+
MAMPs	Chemotaxis protein cheA	+	+	+	+
	Chemotaxis protein cheW	+	+	+	+
	Chemotaxis protein cheY	+	+	+	+
	Chemotaxis response regulator protein-glutamate cheB	+	+	−	+
Type III effectors	*XopAE*	+	+	−	−
	*XopK*	+	+	−	−
	*Xop+*	+	+	−	−
	*XopAL2*	+	+	−	−
	*HopG1*	+	−	−	−
	*HopN1*	+	+	−	−
	*HopV1*	+	+	−	−
	*HopS1*	+	−	−	−
	*HopO1*	+	−	−	−
	*HopT1*	+	−	−	−
	*HopB1*	−	−	+	+
	*RipQ*	+	+	−	−
	*RipTPS*	+	+	−	−
	*AvrE1*	−	+	−	+
Siderophores	Pyochelin	−	−	+	+
	Pyoverdine^*^	−	−	−	+
	Pseudobactin^*^	−	−	−	+
	Hemophore	−	−	−	+
	Arthrobactin	−	−	−	+
Phytohormones	IAA2	−	−	+	+
	Salicylic acid	−	−	−	+
	Salicylic hydroxylase	−	−	−	+
Biofilm	Phosphoglucomutase protein yhxB	−	−	+	+

*+, The complete gene cluster for the genetic factor is present in the genome; −, The gene cluster for the genetic factor was not found or incomplete*.

* *According to PIFAR nomenclature pyoverdine and pseudobactin are considered different siderophores*.

“PIFAR” also searches for putative T3Es by using three databases of well-known bacterial pathogens like *P. syringae* (http://pseudomonas-syringae.org/), *Ralstonia solanacearum* (https://iant.toulouse.inra.fr/bacteria/annotation/cgi/ralso.cgi) and *Xanthomonas* spp. (http://www.xanthomonas.org/t3e.html). The first T3E was identified because its presence led to a hypersensitive response in resistant plants (Staskawicz et al., 1984). Many relevant plant diseases are caused by bacterial pathogens that deliver effector proteins into the eukaryotic host cell by using the T3SS (e.g., Lindeberg et al., 2012), thereby suppressing the plant defense response (Boch, 2009). Twelve and nine T3Es previously described in the pathogenic bacteria *P. syringae* (*Hop* or *AvrE1*; (Baltrus et al., 2011)), *R. solanacearum* (*Rip*; Poueymiro and Genin, 2009; Mukaihara et al., 2010) and *Xanthomonas* (*Xop*; White et al., 2009) that modulate host responses, enabling successful infection and multiplication in plants (Zhou et al., 2008; Guo et al., 2009) were identified in PIC25 and PIC105, respectively (Table 5). In contrast, only one to two T3Es were predicted for PICF141 (*HopB*) and PICF7 (*HopB* and *AvrE1*). While T3Es have been reported mostly in pathogenic bacteria, they have been described in beneficial bacteria as well. For instance, two T3Es identified in *P. fluorescens* Q8r1-96, a strain responsible for the suppressiveness of agricultural soils to take-all disease of wheat, are encoded by the ortholog genes *hopAA1-1* and *hopM1* present in the pathogenic bacterium *P. syringae* (Mavrodi et al., 2011).

Differences were also found for gene clusters putatively encoding for biofilm, siderophores and hormones production (Table 5 and Supplementary Table S10). Complete gene clusters for these traits were only identified in strains of the *P. fluorescens* group that were the only ones to show positive for pyoverdine production (data not shown). Nevertheless, genes coding for siderophore biosynthesis, regulation, and transport were annotated in the genomes of PIC25 and PIC105. While both strains were positive for siderophore activity (Table 3), production of the major siderophore pyoverdine was not detected (data not shown) in agreement with data from “PIFAR” analysis. Therefore, siderophore(s) other than pyoverdine must be produced by these strains. It should be mentioned that “T346hunter” (for secretion systems) and “PIFAR” (for plant-microbe genetic factors) web-based tools only report positive matches for (nearly) complete gene clusters, and that factors defined by several genes are reported only if at least 90% of such genes are found within a given bacterial genome (Martínez-García et al., 2015a, 2016).

### Root colonization ability of novel BCAs from the olive rhizosphere

An indispensable prerequisite for a BCA to succeed in biocontrol is the efficient colonization of the specific niche where it will be applied. In the particular case of beneficial *Pseudomonas* spp. traits involved in rhizosphere and/or root colonization have been studied and reviewed in detail (Mercado-Blanco, 2015; Pizarro-Tobías et al., 2015). For instance, *P. chlororaphis* PCL1391 mutants impaired in root colonization have been shown to lose their biocontrol effectiveness against *Fusarium oxysporum* f. sp. *radicis-lycopersici* in tomato plants (Chin-A-Woeng et al., 2000). Since evaluation of colonization ability of the olive rhizobacteria here studied pose some difficulties due to intrinsic characteristics of woody plant roots (e.g., their large biomass, complicated anatomy, longevity), and experiments with them are usually time consuming (Cazorla and Mercado-Blanco, 2016), tests were carried out using maize as model plant. By doing so, homogeneity of the plant material, effective seeds sterilization, rapid plant growth, and score of reliable results in a short period of time (15 days) were obtained (Roca et al., 2013).

**Figure 3:**
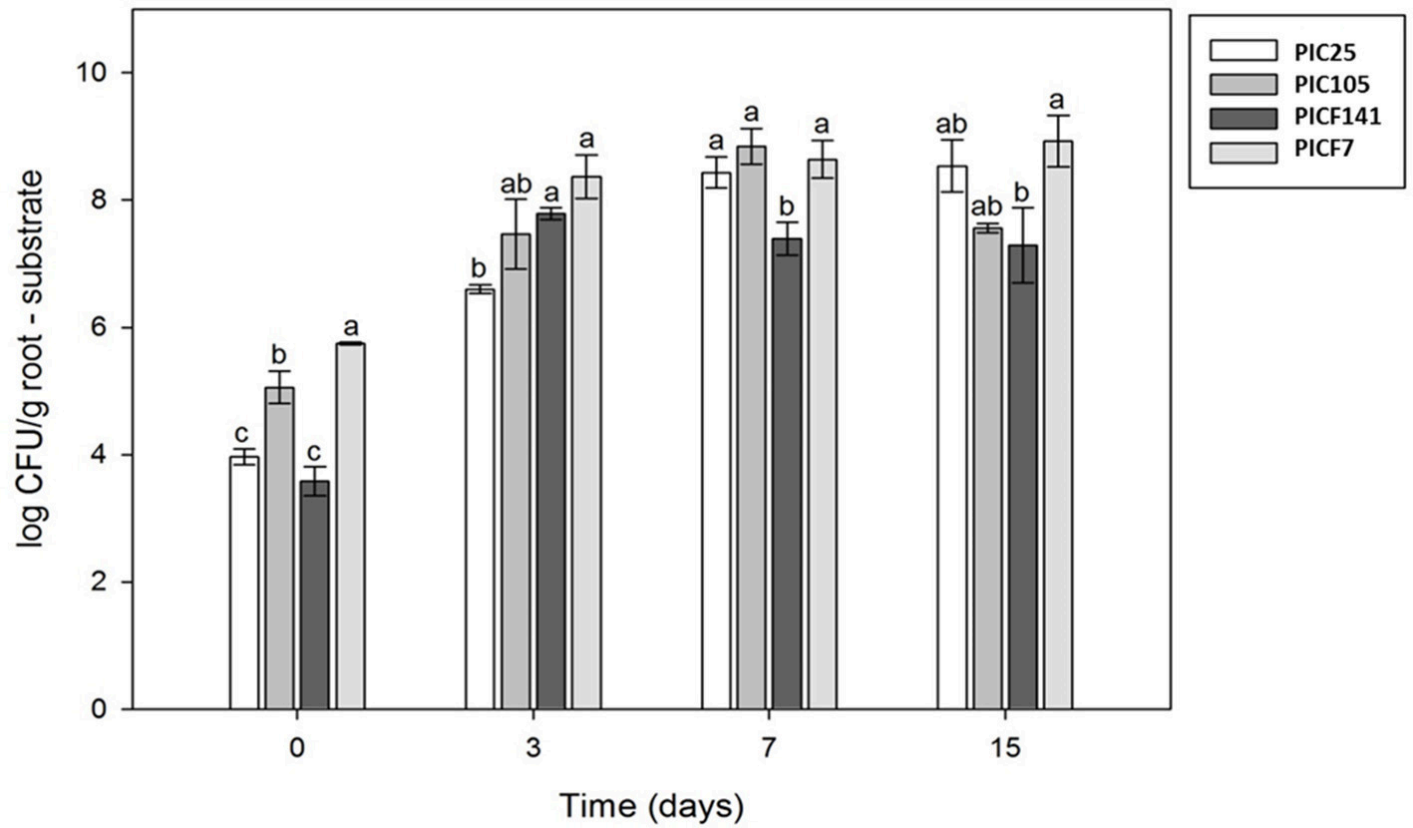
Maize roots colonization ability of the olive rhizobacteria strains *Pseudomonas* sp. PIC25, *Pseudomonas indica* PIC105 and *Pseudomonas* sp. PICF141. Mean number of bacterial cells strongly adhered to maize roots (1 g) are expressed as log CFU/seed at 0, 3, 7, and 15 days after inoculation. Error bars represent the standard deviations (*n* = 3). Different letters indicate significant (*P* < 0.05) differences between treatments in the same sampling day. *Pseudomonas fluorescens* PICF7 was used as reference.

Results from maize root colonization assays showed an increase in the number of bacterial cells attached to the roots for all strains at 3 days after inoculation (DAI), although differences were found (Figure 3 and Supplementary Figure S2). Indeed, significantly lower colonizing efficiency at this time point was evident for strain PIC25, compared to that showed by strains PICF141 and PICF7. Strain PIC105 displayed intermediate root colonization efficiency (Figure 3). However, at 7 DAI, strain PICF141 was the least effective in maize root colonization (around 10^7^ CFU/g root), in contrast to strains PIC25, PIC105, and PICF7 (above 10^8^ CFU/g root). At the end of the assay (15 DAI), the latter three olive rhizobacteria did not show significant differences in their colonization abilities, even though relevant differences on the presence of adhesion factors in their genomes were found (Table 5 and Supplementary Table S10). Moreover, despite the fact that strains PICF141 and PICF7 showed very similar adhesion factors profiles, strain PICF7 colonized significantly better maize roots than strain PICF141 at 7 and 15 DAI (Figure 3). This suggests that other factors involved in adhesion and colonization must be involved to explain the observed differences. Overall, these results indicate that all strains studied present good colonization ability of roots, and that the slight differences found along time do not seem to be crucial to compromise their biocontrol effectiveness, particularly in the case of strain PICF141 that displayed the best biocontrol performance.

## Conclusions

**Figure 4:**
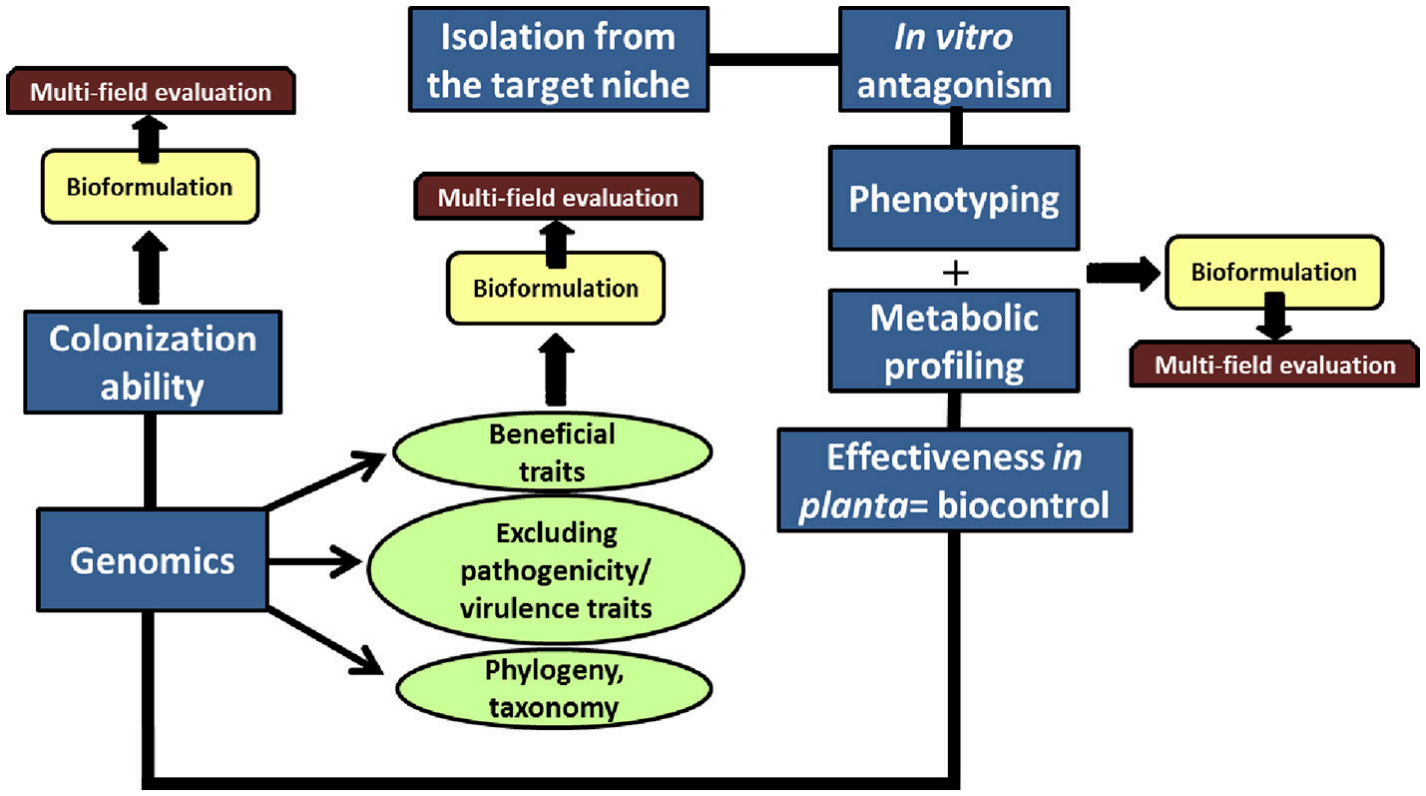
Flowchart of actions to isolate, identify and characterize novel and effective BCAs. This was the strategy followed in this study using the olive root/rhizosphere as target, but it is proposed to be implemented in similar ecological niches.

This study provides a fairly comprehensive approach to identify, characterize, and evaluate new BCAs (Figure 4). By implementing this set of sequential actions we succeeded in identifying three *Pseudomonas* spp. strains, indigenous from the olive rhizobacteria and effective against the D pathotype of *V. dahliae* under non-gnotobitic conditions. Among them, strain PICF141 was the most promising BCA. Therefore, we demonstrated that young plants propagated in nurseries are already an important source of beneficial microorganisms that can be used as biocontrol tools within an integrated management strategy of VWO.

Identification of biocontrol bacteria should not be exclusively based on their performance *in vitro*. Repeated *in planta* experiments conducted under non-gnotobiotic conditions must always be performed to overcome the potential biases usually obtained from antagonism tests carried out under very specific (and artificial) growing conditions. Indeed, *in vitro* experiments showed that strain PIC105 was the most effective to antagonize different olive pathogens. However, *in planta* assays demonstrated strain PICF141 as the most effective against VWO, with a comparable performance to that observed for the well-known BCA *P. fluorescens* PICF7.

The MLSA here performed allowed to assign strains PIC25 and PIC105 to the *P. aeruginosa* group and strain PICF141 to the *P. mandelii* subgroup within the *P. fluorescens* group. Moreover, based on our data we identified strain PIC105 as *P. indica*. It is the first time that a *P. indica* representative is described as a BCA. Full genome sequencing enabled us to acquire valuable information regarding to beneficial traits involved in key aspects like colonization, plant growth promotion and biocontrol abilities of these strains. Besides, *in silico* analyses were useful to confirm the absence of undesirable traits that could compromise the future use of formulations based on these bacterial strains.

Further in-depth insights on specific metabolic and phenotypic abilities of these strains will be valuable for the future development of effective formulations, which can be based on a single strain or tailored consortia. Additionally, it remains to be assessed whether these newly-identified strains are able to endure and protect olive plants under field conditions.

## Author contributions

JM-B conceived the study. All authors participated in the experimental design. CG-LC, DR-R, PP-T, AV-C, JN, AR, and JM-B performed in planta bioassays and conducted experiments aimed to identify and characterize bacterial strains. PP-T, JN and AR conducted root colonization experiments. DR-R and AV-C carried out *in vitro* antagonism tests. GL and JT performed bioinformatics analyses. CG-L and JM-B wrote the article. AR, PP-T, and GL made direct contribution to the final manuscript. All authors have approved the final version.

### Conflict of interest statement

The authors declare that the research was conducted in the absence of any commercial or financial relationships that could be construed as a potential conflict of interest.

## Footnotes

^1^http://www.fao.org/faostat/en/#data/QC
