# Supplementary material for: Indigenous Pseudomonas spp. Strains from the Olive (Olea europaea L.) Rhizosphere as Effective Biocontrol Agents against Verticillium dahliae: From the Host Roots to the Bacterial Genomes
Source: Front Microbiol. 2018 Feb 23;9:277. doi: 10.3389/fmicb.2018.00277 (PMC5829093; doi:10.3389/fmicb.2018.00277)
Supplement: Supplementary file 1 [file Table1.docx]

**Supplementary Table S1.** Primer sequences and PCR conditions used for amplification of genes associated with biological control and plant growth promotion abilities.

| **Gene** | **Product** | **Properties** | **Primer pair** | **Amplicon**  **size (bp)** | **PCR Conditions** | **References for PCR conditions** | **References for compound production in *P. protegens* Pf5** |
| --- | --- | --- | --- | --- | --- | --- | --- |
| ***DAPG*** | 2,4-Diacetylphloroglucinol | Antifungal | Phl2a:  5'-GAGGACGTCGAAGACCACCA-3'  Phl2b:  5'-ACCGCAGCATCGTGTATGAG-3' | 745 | 95°C for 4 min, 30 cycles of 95°C for 30 s, 54°C for 45 s and 72°C for 1 min; 72°C for 10 min. | Modified from Mavrodi *et al*., 2001 | Nowak-Thompson *et al*., 1994  Paulsen *et al*., 2005 |
| ***PRN*** | Pyrrolnitrin | Antifungal | Prncf:  5'-CCACAAGCCCGGCCAGGAGC-3'  Prncr:  5'-GAGAAGAGCGGGTCGATGAAGCC-3' | 720 | 95°C for 3 min, 30 cycles of 95°C for 1 min, 58°C for 1 min and 72°C for 1 min; 72°C for 10 min. | Modified from Mavrodi *et al*., 2001 | Nowak-Thompson *et al*., 1994  Paulsen *et al*., 2005 |
| ***PLT*** | Pyoluteorin | Antifungal | PltBf:  5'-CGGAGCATGGACCCCCAGC-3'  PltBr:  5'-GTGCCCGATATTGGTCTTGACCGAG-3' | 792 | 95°C for 4 min; 30 cycles of 95°C for 30s, 58°C for 45 sand 72°C for 1 min; 72°C for 10 min. | Modified from Mavrodi *et al*., 2001 | Nowak-Thompson *et al*., 1994  Paulsen *et al*., 2005 |
| ***hcnBC*** | Hydrogen cyanide (HCN) | Antifungal | Aca:  5'-ACTGCCAGGGGCGGATGTGC-3'  Acb:  5'-ACGATGTGCTCGGCGTAC-3' | 587 | 95°C for 3 min, 30 cycles of 94°C for 30 s, 54°C for 45s and 72°C for 1 min ; 72°C for 10 min. | Modified from Ramette *et al*., 2003 | Kraus *et al.*, 1992  Paulsen *et al*., 2005 |
| ***rzxB*** | Rhizoxin | Antifungal/  Antibiotic | 2989_5' ENTR:  5´-CACCTCTACAATCACCAGGGCG-3´  2989_3' ENTR:  5´-TTCGCCGTCGATGGAACC-3´ | 1,394 | 94°C for 3 min, 30 cycles of 94°C for 30 s, 54°C for 1 min and 72°C for 1 min; 72°C for 10 min. | Adapted from Loper *et al*., 2008  Roca, A. unpublished | Loper *et al*., 2008 |
| ***rzxB***  ***(flanking región)*** | Rhizoxin | Antifungal/  Antibiotic | 2989_5' OUT:  5´-ACTGGGCGAACGGATATG-3´  2989_3' OUT:  5´-GAACTTCGCCGTACACGC-3´ | 1,637 | 94°C for 3 min, 30 cycles of 94°C for 30 s, 52°C for 1 min and 72°C for 1 min; 72°C for 10 min. | Adapted from Loper *et al*., 2008  Roca, A. unpublished | Loper *et al*., 2008 |
| ***PFL_2018*** | Pyocin | Bactericide | PFL_2018 A:  5´-ATGACCATCACCGCGCAGCAGCTGC-3´  PFL_2018 B:  5´-TCACGCCAGCACCTTCAATGCACGC-3´ | 552 | 95°C for 4 min, 30 cycles of 95°C for 30 s, 61°C for 45 sand 72°C for 1 min ; 72°C for 10 min. | Roca, A. unpublished | Parret *et al*., 2005 |
| ***FitD1*** | *P. fluorescens* insecticidal  toxin | Insecticide | Fit D 1A:  5´-atggcttttatgtccaaggacttca-3´  FitD 1B:  5´-TCAGCCGTCTCGGAAGAT-3´ | 2,160 | 95°C for 4 min, 30 cycles of 95°C for 30 s, 53°C for 1 min and 72°C for 1 min; 72°C for 10 min. | Roca, A. unpublished | Loper *et al*., 2016 |
| ***FitD2*** | *P. fluorescens* insecticidal  toxin | Insecticide | Fit D 2A:  5´-gcccacagtaactggtggaaaaacc-3´  FitD 2B:  5´-TCAGGTCAGTGAAGGCACCAGCACC-3´ | 1,395 | 95°C for 4 min, 30 cycles of 95°C for 30 s, 58°C for 1 min and 72°C for 1 min; 72°C for 10 min. |  | Parret *et al*., 2005 |
| ***PFL_1227*** | Pyocin | Bactericide | PFL_1227 A:  5´-ATGCCAATAACCGAAGAACAACTGC-3  PFL_1227 B:  5´-TCAAACCACCGGCAAACACAGCACC-3´ | 564 | 95°C for 4 min, 30 cycles of 95°C for 30 s, 55°C for 45 s and 72°C for 1 min ; 72°C for 10 min. | Roca, A. unpublished | Loper *et al*., 2016 |

**Supplementary Table S2.** Biochemical properties, utilization of carbon sources, chemical sensibilities for strains PIC25, PIC105, PICF141 and the reference BCA PICF7.

|  | **Strains** | | | |  | | **Strains** | | | | | |  |
| --- | --- | --- | --- | --- | --- | --- | --- | --- | --- | --- | --- | --- | --- |
| **Carbon source**  **utilization assays** | PICF7 | PIC25 | PIC105 | PICF141 | **Chemical sensitivity assays** | | PICF7 | | PIC25 | PIC105 | | PICF141 | |
| Dextrin | - | + | + | - | pH 6 | | + | | + | + | | + | |
| D-Maltose | - | - | - | + | pH 5 | | + | | +/- | + | | + | |
| D-Trehalose | + | - | - | + | 1% NaCl | | + | | + | + | | + | |
| D-Cellobiose | - | - | - | - | 4% NaCl | | - | | +/- | + | | - | |
| Gentiobiose | - | - | - | - | 8% NaCl | | - | | - | - | | - | |
| Sucrose | - | + | + | + | 1% Sodium Lactate | | + | | + | + | | + | |
| D-Turanose | - | - | - | + | Fusidic Acid | | + | | + | + | | - | |
| Stachyose | - | - | - | - | D-Serine | | + | | - | - | | - | |
| D-Raffinose | - | - | - | - | Troleandomycin | | + | | + | + | | + | |
| α-D-Lactose | - | - | - | - | Rifamycin SV | | + | | + | + | | + | |
| D-Melibiose | - | - | - | - | Minocycline | | - | | - | - | | - | |
| β-Methyl-D- Glucoside | - | - | - | - | Lincomycin | | + | | + | + | | + | |
| D-Salicin | - | - | - | - | Guanidine HCl | | + | | + | + | | + | |
| N-Acetyl-D- Glucosamine | * | - | - | - | Niaproof 4 | | + | | + | + | | + | |
| N-Acetyl-β-D- Mannosamine | - | - | - | - | Vancomycin | | + | | + | + | | + | |
| N-Acetyl-D- Galactosamine | - | - | - | - | Tetrazolium Violet | | + | | + | + | | + | |
| N-Acetyl Neuraminic Acid | - | - | - | - | Tetrazolium Blue | | + | | + | + | | + | |
| α-D-Glucose | + | + | + | + | Nalidixic Acid | | + | | +/- | + | | - | |
| D-Mannose | + | - | - | + | Lithium Chloride | | - | | +/- | + | | - | |
| D-Fructose | + | + | + | + | Potassium Tellurite | | + | | +/- | + | | + | |
| D-Galactose | + | - | - | + | Aztreonam | | + | | +/- | + | | + | |
| 3-Methyl Glucose | - | - | - | - | Sodium Butyrate | | - | | +/- | - | | - | |
| D-Fucose | * | - | - | - | Sodium Bromate | | - | | - | - | | - | |
| L-Fucose | * | - | - | - |  | |  | |  |  | |  | |
| L-Rhamnose | - | - | - | - |  | |  | |  |  | |  | |
| Inosine | * | - | - | - |  | |  | |  |  | |  | |
| D-Serine | + | + | - | - |  | |  | |  |  | |  | |
| D-Sorbitol | + | - | - | - |  | |  | |  |  | |  | |
| D-Mannitol | + | + | + | - |  | |  | |  |  | |  | |
| D-Arabitol | * | - | - | - |  | |  | |  |  | |  | |
| myo-Inositol | + | - | + | + |  | |  | |  |  | |  | |
| Glycerol | + | + | + | + |  | |  | |  |  | |  | |
| D-Glucose- 6-PO4 | - | - | - | - |  | |  | |  |  | |  | |
| D-Fructose- 6-PO4 | * | + | - | - |  | |  | |  |  | |  | |
| D-Aspartic Acid | + | - | - | - |  | |  | |  |  | |  | |
| Gelatin | - | - | - | - |  | |  | |  |  | |  | |
| Glycyl-L-Proline | - | + | + | - |  | |  | |  |  | |  | |
| L-Alanine | + | + | + | * |  | |  | |  |  | |  | |
| L-Arginine | + | + | + | + |  | |  | |  |  | |  | |
| L-Aspartic Acid | + | + | + | + |  | |  | |  |  | |  | |
| L-Glutamic Acid | + | + | + | + |  | |  | |  |  | |  | |
| L-Histidine | + | + | + | - |  | |  | |  |  | |  | |
| L-Pyroglutamic Acid | + | + | + | - |  | |  | |  |  | |  | |
| L-Serine | + | + | + | + |  | |  | |  |  | |  | |
| D-Serine |  | + | - | - |  | |  | |  |  | |  | |
| Pectin | - | + | + | - |  | |  | |  |  | |  | |
| D-Galacturonic Acid | + | + | + | - |  | |  | |  |  | |  | |
| L-Galactonic Acid Lactone | - | + | + | - |  | |  | |  |  | |  | |
| D-Gluconic Acid | + | + | + | - |  | |  | |  |  | |  | |
| D-Glucuronic Acid | + | + | + | - |  | |  | |  |  | |  | |
| Glucuronamide | * | + | + | - |  | |  | |  |  | |  | |
| Mucic Acid | + | + | + | + |  | |  | |  |  | |  | |
| Quinic Acid | + | - | + | + |  | |  | |  |  | |  | |
| D-Saccharic Acid | + | + | + | + |  | |  | |  |  | |  | |
| p-Hydroxy- Phenylacetic Acid | + | - | - | - |  | |  | |  |  | |  | |
| Methyl Pyruvate | - | + | + | - |  | |  | |  |  | |  | |
| D-Lactic Acid Methyl Ester | - | - | - | - |  | |  | |  |  | |  | |
| L-Lactic Acid | + | + | + | - |  | |  | |  |  | |  | |
| Citric Acid | + | + | + | + |  | |  | |  |  | |  | |
| α-Keto-Glutaric Acid | + | + | + | - |  | |  | |  |  | |  | |
| D-Malic Acid | - | + | + | - |  | |  | |  |  | |  | |
| L-Malic Acid | + | + | + | + |  | |  | |  |  | |  | |
| Bromo-Succinic Acid | - | + | + | - |  | |  | |  |  | |  | |
| Tween 40 | - | + | + | - |  | |  | |  |  | |  | |
| γ-Amino-Butryric Acid | + | + | + | + |  | |  | |  |  | |  | |
| α-Hydroxy- Butyric Acid | - | - | - | - |  | |  | |  |  | |  | |
| β-Hydroxy-D,L- Butyric Acid | + | + | + | - |  | |  | |  |  | |  | |
| α-Keto-Butyric Acid | - | + | - | - |  | |  | |  |  | |  | |
| Acetoacetic Acid | - | - | - | - |  | |  | |  |  | |  | |
| Propionic Acid | + | + | + | - |  | |  | |  |  | |  | |
| Acetic Acid | + | + | + | + |  | |  | |  |  | |  | |
| Formic Acid | - | - | - | - |  |  | |  | |  |  | | |

+, ability to use this carbon source or no sensitivity to the inhibitory chemical; –, no ability to use this carbon source or significant sensitivity to the inhibitory chemical; ±, one replicate showed positive result and the other negative and, * weak signal.

**Supplementary Table S3.** General information of the three sequencing projects.

| **MIGS ID** | **Property** | **Term** |
| --- | --- | --- |
| MIGS-31 | Finishing quality | Finished |
| MIGS-28 | Libraries used | PCR-free 550 pb |
| MIGS-29 | Sequencing platforms | Illumina Miseq |
| MIGS-31.2 | Fold coverage | 244 x (PIC25)  255x (PIC105)  157x (PICF141) |
| MIGS-30 | Assemblers | Megahit v1.0.3 |
| MIGS-32 | Gene calling method | NCBI Prokaryotic Genome Annotation Pipeline |
|  | Locus Tag | PIC25  PIC105  PICF141 |
|  | Genbank ID | MUJY00000000 (PIC25)  MUNN00000000 (PIC105)  MUNM00000000 (PICF141) |
|  | GOLD ID | - |
|  | BIOPROJECT | PRJNA369069 |
|  | NCBI taxon ID | 286 |
|  | Project relevance | Plant-bacteria interaction,  Agricultural, Environmental |

MIGS, minimum information about a genome sequence (Field et al., 2008).

**Supplementary Table S4.** Number of genes associated with GO functional categories.

|  | **PIC25** | | **PIC105** | | **PICF141** | |  |
| --- | --- | --- | --- | --- | --- | --- | --- |
| **Code** | **Value** | **% of total ^a^** | **Value** | **% of total ^a^** | **Value** | **% of total ^a^** | **Description** |
| A | 3 | 0,094 | 2 | 0,062 | 1 | 0,029 | RNA processing and modification |
| C | 189 | 5,893 | 191 | 5,917 | 208 | 6,024 | Energy production and conversion |
| B | 1 | 0,031 | 1 | 0,031 | 1 | 0,029 | Chromatin structure and dynamics |
| E | 312 | 9,729 | 345 | 10,688 | 418 | 12,105 | Amino acid transport and metabolism |
| D | 31 | 0,967 | 28 | 0,867 | 33 | 0,956 | Cell cycle control, cell division, chromosome partitioning |
| G | 153 | 4,771 | 164 | 5,081 | 186 | 5,387 | Carbohydrate transport and metabolism |
| F | 76 | 2,370 | 75 | 2,323 | 74 | 2,143 | Nucleotide transport and metabolism |
| I | 175 | 5,457 | 175 | 5,421 | 175 | 5,068 | Lipid transport and metabolism |
| H | 162 | 5,051 | 167 | 5,173 | 172 | 4,981 | Coenzyme transport and metabolism |
| K | 220 | 6,860 | 225 | 6,970 | 285 | 8,254 | Transcription |
| J | 219 | 6,829 | 218 | 6,753 | 227 | 6,574 | Translation, ribosomal structure and biogenesis |
| M | 167 | 5,207 | 162 | 5,019 | 187 | 5,416 | Cell wall/membrane/envelope biogenesis |
| L | 131 | 4,085 | 113 | 3,501 | 116 | 3,359 | Replication, recombination and repair |
| O | 127 | 3,960 | 128 | 3,965 | 146 | 4,228 | Posttranslational modification, protein turnover, chaperones |
| N | 119 | 3,711 | 113 | 3,501 | 76 | 2,201 | Cell motility |
| Q | 110 | 3,430 | 107 | 3,315 | 111 | 3,215 | Secondary metabolites biosynthesis, transport and catabolism |
| P | 170 | 5,301 | 171 | 5,297 | 201 | 5,821 | Inorganic ion transport and metabolism |
| S | 112 | 3,492 | 113 | 3,501 | 111 | 3,215 | Function unknown |
| R | 288 | 8,980 | 300 | 9,294 | 330 | 9,557 | General function prediction only |
| U | 63 | 1,964 | 65 | 2,014 | 37 | 1,072 | Intracellular trafficking, secretion, and vesicular transport |
| T | 263 | 8,201 | 255 | 7,900 | 271 | 7,848 | Signal transduction mechanisms |
| W | 20 | 0,624 | 18 | 0,558 | 10 | 0,290 | Extracellular structures |
| V | 78 | 2,432 | 76 | 2,354 | 60 | 1,738 | Defense mechanisms |
| Y | 0 | 0,000 | 0 | 0,000 | 0 | 0,000 | Nuclear structure |
| X | 18 | 0,561 | 16 | 0,496 | 17 | 0,492 | Mobilome: prophages, transposons |
| Z | 0 | 0,000 | 0 | 0,000 | 0 | 0,000 | Cytoskeleton |

^a^The total is based on the total number of protein coding genes in the annotated genome.

**Supplementary Table S9**. Secretion systems identified in the genomes of *Pseudomonas* spp. strains PIC25, PIC105, PICF141 and PICF7.

| **Secretion systems** | **PIC25** | **PIC105** | **PICF141** | **PICF7** |
| --- | --- | --- | --- | --- |
| T3SS1 flagellar | + | + | + | - |
| T3SS1 non-flagellar | + | - | - | + |
| T3SS2 flagellar | + | + | + | + |
| T3SS2 non-flagellar | - | + | - | - |
| T3SS3 flagellar | + | + | + | + |
| T4SS1 | + | + | - | - |
| T4SS2 | + | - | - | - |
| T4SS3 | + | - | - | - |
| T6SS1 | + | + | + | + |
| T6SS2 | + | + | + | + |

Completed secretion systems gene clusters identified using the T346hunter web application (Martínez-García et al., 2015).

**Supplementary Table S10.** Summary of genes identified in the genomes of *Pseudomonas* spp. strains PIC25, PIC105, PICF141 and PICF7 involved in plant-bacteria interaction.

|  | ***Pseudomonas* spp. strains** | | | |
| --- | --- | --- | --- | --- |
|  | **PIC25** | **PIC105** | **PICF141** | **PICF7** |
| **Nº Annotated genes** | 112 | 108 | 116 | 153 |
| Proteases | 2 | 2 | 2 | 2 |
| PCWDEs | 7 | 6 | 1 | 2 |
| Adhesion | 5 | 3 | 10 | 19 |
| Detoxification | 12 | 12 | 16 | 23 |
| EPSs | 13 | 13 | 15 | 14 |
| Metabolism | 12 | 13 | 12 | 12 |
| LPSs | 4 | 4 | 3 | 2 |
| MDRs | 26 | 30 | 33 | 37 |
| Volatiles | 4 | 4 | 2 | 2 |
| Antibiotics | 4 | 3 | 9 | 5 |
| MAMPs | 5 | 8 | 3 | 4 |
| TYPE III effectors | 18 | 10 | 1 | 2 |
| Biofilm | - | - | 1 | 1 |
| Siderophores | - | - | 5 | 23 |
| Phytohormones | - | - | 2 | 5 |

Completed gene clusters involved in plant-bacteria interaction identified using the T346hunter web application (Martínez-García et al., 2016). - , the gene cluster for the genetic factor was not found or incomplete.
