# Supplementary material for: Indigenous Pseudomonas spp. Strains from the Olive (Olea europaea L.) Rhizosphere as Effective Biocontrol Agents against Verticillium dahliae: From the Host Roots to the Bacterial Genomes
Source: Front Microbiol. 2018 Feb 23;9:277. doi: 10.3389/fmicb.2018.00277 (PMC5829093; doi:10.3389/fmicb.2018.00277)

Supplementary figure 1. Phylogenetic tree showing the taxonomic position of the three new *Pseudomonas* spp. strains (arrowed) isolated from the olive rhizobacteria. The tree was inferred by the Neighbor-Joining method, based on the alignment of concatenated partial sequences of the *16S rRNA*, *gyrB*, *atpA*, *nusA*, *recA* and *dnaJ* genes. Bar indicates sequence divergence. Bootstrap values (>95%) based on 1000 re-sampled datasets are shown at branch nodes. *Pseudomonas entomophila* L48 strain was used as out-group.

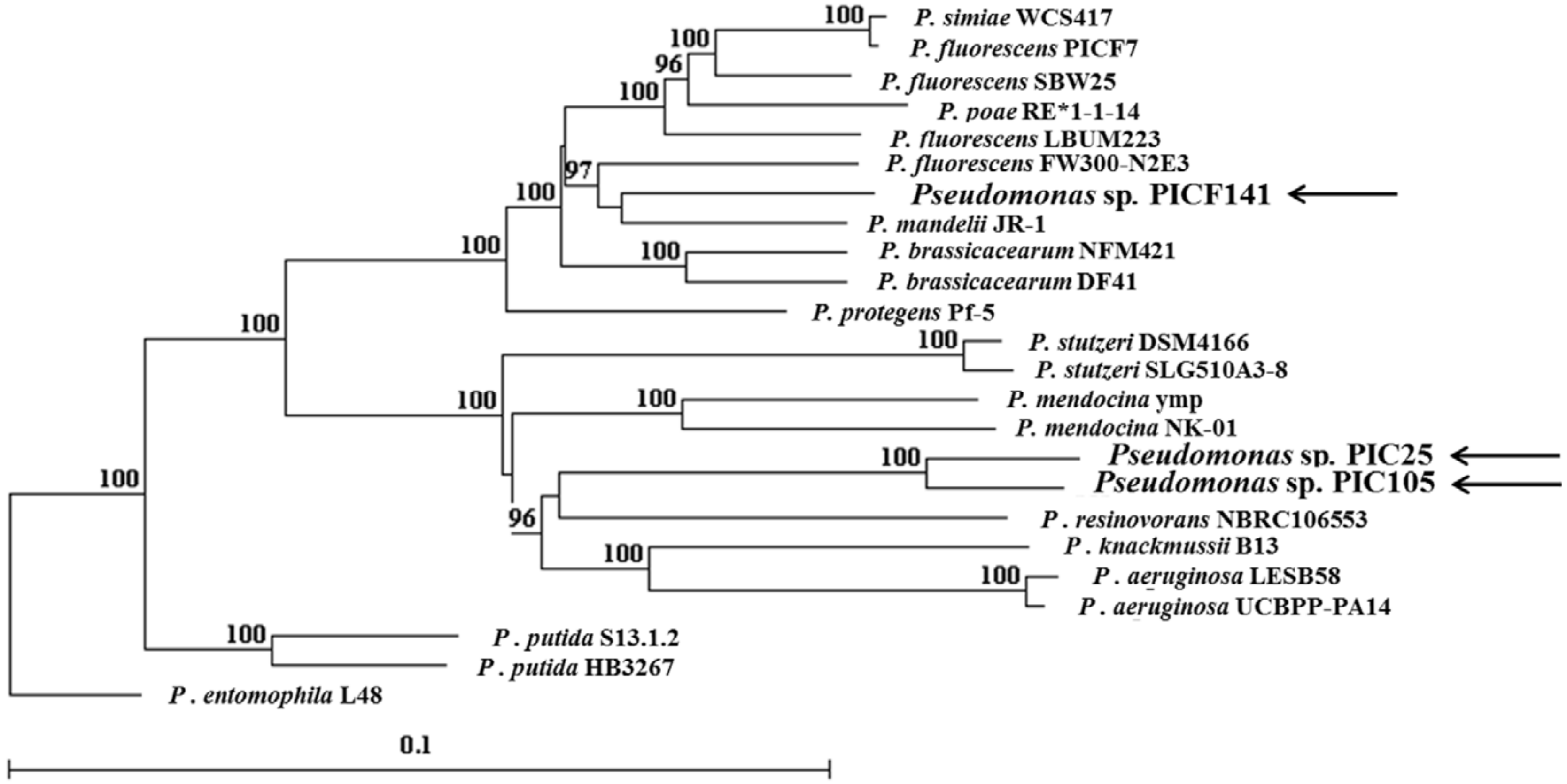

Supplement: Supplementary file 2 [file Image1.PDF]
