# Supplementary material for: Indigenous Pseudomonas spp. Strains from the Olive (Olea europaea L.) Rhizosphere as Effective Biocontrol Agents against Verticillium dahliae: From the Host Roots to the Bacterial Genomes
Source: Front Microbiol. 2018 Feb 23;9:277. doi: 10.3389/fmicb.2018.00277 (PMC5829093; doi:10.3389/fmicb.2018.00277)

Supplementary figure 2. Maize roots colonization ability of the olive rhizobacteria strains PIC25, PIC105, PIC141 and PICF7. Results are expressed as percentages of the increase of each bacterial population with respect to that scored at inoculation time (T=0 days). Strain PIC25 (black bars); strain PIC105 (light grey bars); strain PIC141 (dark grey bars); and strain PICF7 (white bars, used as a reference). See main text for details and Figure 3.

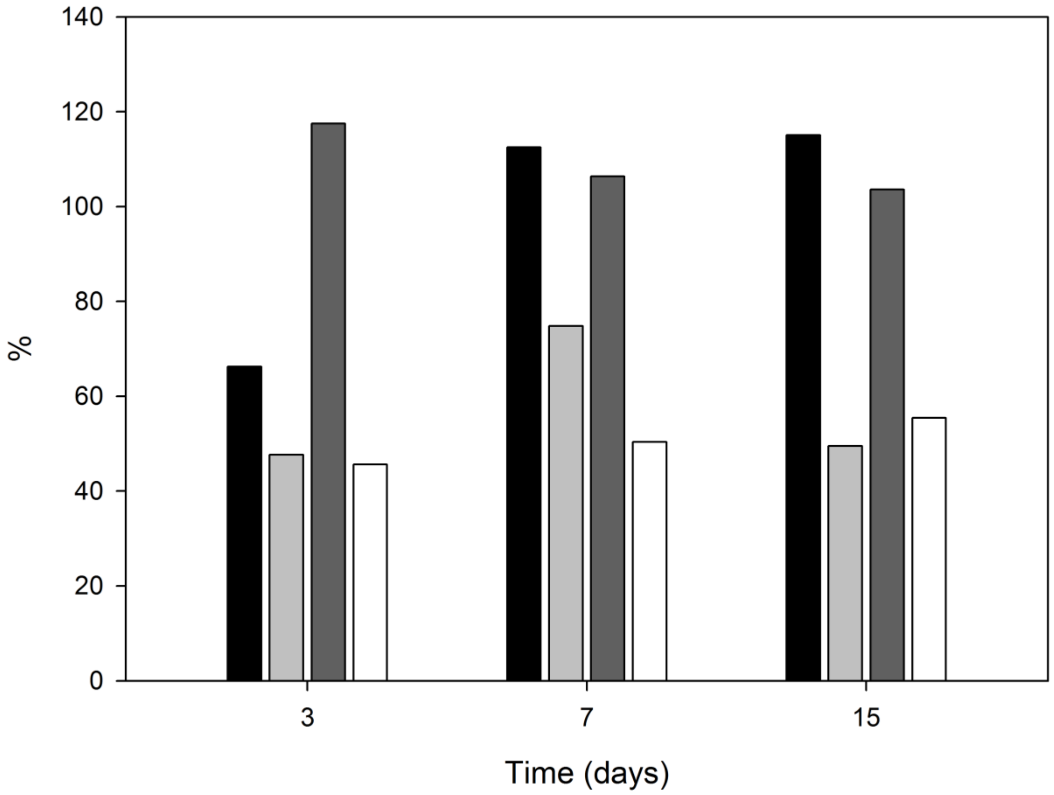

Supplement: Supplementary file 3 [file Image2.PDF]
